# Performance of Restricted Mean Survival Time Based Methods and Traditional Survival Methods: An Application in an Oncological Data

**DOI:** 10.1155/2022/7264382

**Published:** 2022-12-28

**Authors:** Qiao Huang, Chong Tian

**Affiliations:** Tongji Medical College, Huazhong University of Science and Technology, China

## Abstract

**Objective:**

To compare restricted mean survival time- (RMST-) based methods with traditional survival methods when multiple covariates are of interest.

**Methods:**

4405 osteosarcomas were captured from Surveillance, Epidemiology, and End Results Program Database. RMST-based methods included group comparison using Kaplan-Meier (KM) method, pseudovalue (PV) regression, and inverse probability of censoring probability (IPCW) regressions with group-specific and individual weights. Log-rank test, Wilcoxon test, Cox regression, and its extension with time-dependent variables were selected as traditional methods. Proportional hazard (PH) assumption and homogeneity of censoring mechanism assumption were assessed. We estimated hazard ratio (HR) and difference in RMST and explored their relationships.

**Results:**

When covariate violated PH assumption, time-varying HR was inconvenient to report as a single value but PH assumption-free RMST allowed to report a single value of difference in RMST. In univariable analyses, using the difference in RMST calculated by KM method as reference, PV regressions (slope = 1.02 and *R*^2^ = 0.98) and IPCW regressions with group-specific weights (slope = 0.98 and *R*^2^ = 0.99) gave more consistent estimation than IPCW with individual weights (slope = 0.31 and *R*^2^ = 0.06), moreover, PV regressions presented more robust statistical power than IPCW regressions with group-specific weights. In multivariable analyses, IPCW regression with group-specific weights was limited when multiple covariates violated homogeneity of censoring mechanism assumption. For covariates met PH assumption, well-fitted logarithmic relationships between HR and difference in RMST estimated by PV regression were observed in both univariable and multivariable analyses (*R*^2^ = 0.97 and *R*^2^ = 0.94, respectively), which supported the robustness of PV regression and possible conversion between the two effect measures.

**Conclusions:**

Difference in RMST is more interpretable than time-varying HR. The performance supports KM method and PV regression to be the preferred ones in RMST-based methods. IPCW regression can be an alternative sensitivity analysis. We encourage adoption of both traditional methods and RMST-based methods to present effects of covariates comprehensively.

## 1. Background

In oncological studies, survival data, involving survival time (until the occurrence of an event of interest) and status (event or censor), are frequently adopted [1]. Features of censoring make survival analyses different and common form of censoring is right censoring. Generally, survival analysis starts from data description and exploration by plotting Kaplan-Meier (KM) curves, and then compares survival curves; finally, univariable and multivariable regressions are performed to obtain effects of one or more factors [2]. When only one factor is of interest (such as intervention group), unadjusted and adjusted effect measure of the factor are reported. When focusing on multiple factors (such as risk factors), unadjusted and adjusted effect measures of each factor are of interest.

Hazard ratio (HR) from Cox proportional hazard regression (referred as Cox regression) is routinely considered as a preferred effect measure, but interpretation of HR may be challenging since it is not an intuitively and clinically summary statistic. Note that a key assumption for Cox regression is the proportional hazards (PH) assumption which requires a constant HR over time [3]. If PH assumption is not satisfied, estimated HR with single value from Cox regression is invalid and incorrect conclusion may be made [4]. Restricted mean survival time (RMST), the area under the survival curve between 0 and a prespecific time (*τ*), has been promoted as an alternative and a better summary statistic for survival data, it is not limited to the PH assumption [5, 6]. RMST can be interpreted in a clinically meaningful perspective. Difference in RMST as an absolute effect presents the length of average gain or lost in life expectancy within prespecific time (*τ*), negative difference means survival time lost (STL) and positive difference means survival time gain (STG). Meanwhile, in consolidated standards of reporting trials (CONSORT) and strengthening the reporting of observational studies in epidemiology (STROBE), relative and absolute effect size are encouraged to be reported together to present a broad picture of effects of covariates [7, 8]. Reporting both HR and difference in RMST in oncological study can be an attractive move.

Simplicity and clinical intuition of RMST promotes development of RMST-based methods. When only one categorical covariate is of interest, unadjusted difference in RMST can be estimated based on Kaplan-Meier curve, and adjusted one is estimated by standardized survival curves. When focusing on multiple factors, RMST based regressions make it feasible to explore independent effects of multiple covariates on RMST. Three regressions had been proposed, pseudovalue (PV) regression and inverse probability censoring weighting (IPCW) regression with group-specified weights and IPCW with individual weights [9, 10]. Homogeneity of censoring mechanism assumption determines the selection.

A large number of survival methods may hinder researchers and lay people from choosing the appropriate method conveniently and effectively. Understanding their performance in a real research and relationship between the two effect measures can facilitate researchers to report. In this study, to explore performance of different methods, we conducted traditional survival methods and RMST-based methods on an oncological data, summarized and compared the results, and explored relationship between HR and difference in RMST. Finally, we summarized these methods and drew a flowchart.

## 2. Methods

### 2.1. Oncological Data

The Surveillance, Epidemiology, and End Results (SEER) Program Database was accessed and Osteosarcoma cases with complete data on status and survival time from 1970s to 2000s was captured. Cases of those who died or censored at time 0 were excluded from the analysis because of different processing strategies in general statistical software.

Patients' demographic covariates included age, sex, the year of diagnosis, and race. Age was categorized into >60 years and ≤60 years, and race were classified as white and others. Data on tumor included tumor size, extension of disease, and American Joint Committee on Cancer for staging (AJCC). Tumor size was categorized into >100 mm, <100 nm, and “UNKNOWN”. Data on treatment included surgery, chemotherapy, and radiation, all of which were “yes” and “no”. Overall survival was the outcome of interest, including survival status (dead for all causes or alive) and survival time in months.

### 2.2. Statistical Methods

#### 2.2.1. Data Description

Characteristics of participants were descripted for categorical variables (frequencies and percentages) and continuous variables (means with SD or medians with interquartile range). Median of follow-up months and number of death were calculated.

#### 2.2.2. Assumption Assessment

PH assumption and Homogeneity of censoring mechanism assumption were appraised. Three methods existed for PH assumption assessment: (1) graphical assessment (KM curves and ln (-ln(S(t))) vs. ln(t) Curves); (2) significance test for the interaction of covariate ^∗^log(t); and (3) global good of fitness by plotting and testing association between ranked survival time and Schoenfeld residuals [11]. In this study, large sample size made *P* values sensitive to little departure from PH assumption, graphics assessment was selected. The homogeneity of censoring mechanism assumption was evaluated by KM curve or log-rank test, in which the censoring parameter was specified as 1 instead of 0 (0 indicating a censored time and the value 1 indicating an event time). A substantially separated KM curves or a small *P* value from log-rank test supported the violation of the homogeneity of censoring mechanism.

#### 2.2.3. Traditional Survival Methods

Log-rank test is the most powerful under PH assumption. Non-PH patterns required alternative methods, including Wilcoxon test (Breslow test), Tarone-Ware test, and combination tests. In this study, log-rank test, Wilcoxon test, and Cox regressions, considered as traditional methods, were performed to estimate *P* value and HRs. All covariates were of interest. When included covariates violated the PH assumption, we conducted extended Cox regression with time-dependent variable (covariate × log_e_ (time)) instead of stratified Cox regression.

#### 2.2.4. RMST-Based Methods

In RMST based methods, difference in RMST was selected as effect measure. Group comparison based on KM method and 2 kinds of RMST-based regressions were conducted. Coefficients estimated from RMST-based regressions were the difference in RMST. *τ* specified in group comparison was the smallest value of the longest follow-up time across groups minus 5 months. *τ* specified in univariable regression was same as that obtained from group comparison. In multivariable analyses, *τ* was fixed at 480 months (24years) for clinical comparison.

RMST-based regressions consisted of PV and IPCW regressions, which use generalized linear modeling techniques to directly model the RMST. In PV regression, jackknife leave-one-out estimation is employed to generate pseudovalues [12]. Firstly, the area under the Kaplan-Meier curve up to time *τ* is estimated as $\hat{\theta }$ on the complete data with *n* subjects, then remove *i*-th subject and repeat the above estimation to get leave-one-out estimator as $\hat{\theta^{-i}}$. Finally, the *i*-th pseudovalues of $\hat{\theta_{i}}$ can be calculated based on the difference between the complete sample and the leave-one-out estimator: $\hat{\theta_{i}}=n\hat{\theta }-\left(n-1\right)\hat{\theta^{-i}}$. Calculated pseudovalue for each subject is used to as dependent variable to model effects of covariates on RMST [10]. In IPCW regression, conditional probability of remaining uncensored until time *t* is calculated as weight for each participant and inverse of estimated weights are included into iterative estimation of coefficients of IPCW regression [9]. Kaplan-Meier method is used to determine censoring distribution (modeling censoring mechanism). It assumes that all participants share the same censoring distribution, namely homogeneity of censoring mechanism assumption. IPCW regression is extended into IPCW regression with individual weights and IPCW regression with group-specified weights. When a categorical covariate is of interest and censoring distribution in each level are not the same, it is appropriate to use group-specific weights where censoring mechanism keep homogenous in each group and weights were estimated separately for each group. In multivariable analysis, IPCW regression with group-specified weights was not chosen since only one categorical covariate was allowed to be specified to stratify censoring distribution.

#### 2.2.5. Relationship Exploration

To test consistency in difference in RMST estimated by 4 methods, scatter plots with fitted lines were created, using Kaplan-Meier method as a golden reference. Relationship between HR and difference in RMST was visualized for covariates holding PH assumption. Since HR was a ratio while difference in RMST was a difference, a log transformed nonlinear regression was considered when fitting the relationship (difference in RMST ~ log_e_(HR)).

All analyses were carried out using the SAS software for windows, version 9.4 TS1M6 (SAS Institute Inc, Cary, NC) with a 2-sided significance threshold of *P* < .05. Data visualization were conducted in Microsoft PowerPoint 2016 and R 4.03 (Vienna, Austria) with ggplot2 package.

## 3. Results

4505 patients were extracted from SEER database. 100 patients with 0 survival time were excluded from final analyses. Of 4405, 2389 (54.23%) deaths were documented at the end of the study during a median follow-up of 35 months. The 2 assumptions were assessed (Table 1). Age, year of diagnosis, extend of disease, surgery, and radiation held PH assumption. Age, sex, race, and radiation met homogeneity of censoring mechanism assumption. More details in graphical assessment were collected in supplementary material (available here) (Graphic assessment of Proportional hazard assumption).

The median age of patients was 30 years old, and 19.09% of them were older than 60 years, more than half of patients underwent surgery (77.80%) and chemotherapy (67.17%), while few cases (14.10%) underwent radiation (Table 2). In group comparison, *P* values of the covariates from log-rank test, Wilcoxon test, and unadjusted difference in RMST were similar, except for sex and race. Sex and Race did not satisfy PH assumption, Wilcoxon tests were more appropriate. Survival curves showed no statistical difference between males and females (*P* = 0.058) but statistical difference between white and others (*P* = 0.004). However, difference in RMST showed opposite conclusion, males lived shorter by 24.31 months than females (*P* = 0.004), and no significant difference exist between white and others (*P* = 0.21). Dichotomous age was used as an example to explain HR and RMST. Compared with patients younger than 60 years, hazard rate of patients older than 60 years increased by 282% (HR = 3.82, *P* < 0.001), while their RMST decreased by 146.64 months (*P* < 0.001).

**Figure 1:**
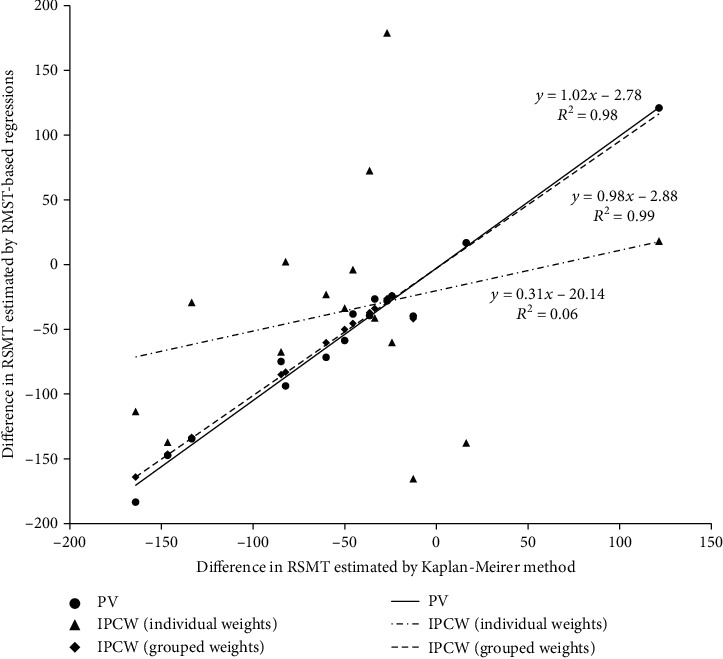
Difference in restricted mean survival time estimated from 4 methods in univariable analyses.

Unadjusted differences in RMST from four methods were summarized into Table 2, and their relationship was visualized in Figure 1. Using difference in RMST based on Kaplan-Meier method as reference, PV regression and IPCW regression with group-specified weights exhibited strongly consistent results (slope = 1.02 and *R*^2^ = 0.98, slope = 0.98 and *R*^2^ = 0.99, respectively), but IPCW regression with individual weights showed inconsistent result (slope = 0.31 and *R*^2^ = 0.06). Even though dichotomous age, sex, race, and radiation that met the homogeneity of censoring mechanism assumption, IPCW regression with individual weights gave less consistent estimates than IPCW regression with group-specified weights. But, IPCW regressions with group-specific weights showed that *P* values for sex, tumor size, and chemotherapy turned to be insignificant, its statistical power decreased.

In multivariable regressions, IPCW regression with group-specified weights could not be conducted when multiple covariates violated homogeneity of censoring mechanism assumption. We performed IPCW regression with individual weights instead with biased weights. Compared with coefficients and *P* values from PV regression, IPCW regression with individual weights presented great differences. For example, difference in RMST of radiation therapy was -45.76 (95% CI -67.87 to -31.99, *P* < 0.001) in PV regression and -6.52 (95% CI -58.84 to 45.80, *P* = 0.807) in IPCW regression with individual weights (Table 3).

**Figure 2:**
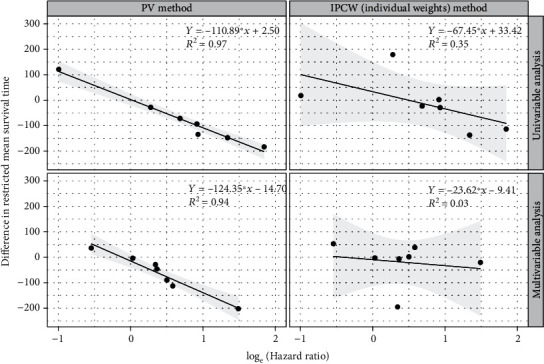
Relationships between log-transformed hazard ratios from Cox PH regressions and differences in RMST from RMST based regressions in both univariable and multivariable regressions.

In Cox regressions, estimated HR of covariate violating the PH assumption is a function of time *e*^(*β*_1_ + *β*_2_∗*t*)^ instead of a single value, so it was presented as “time-varying” instead of value in tables (Tables 2 and 3). For covariates met PH assumption, a stable and well-fitted logarithmic relationships between HR and difference in RMST estimated by PV regression were observed in both univariable and multivariable regressions, difference in RMST = −110.89^∗^log_e_(HR) + 2.50 and *R*^2^ = 0.97, difference in RMST = −124.35^∗^log_e_(HR) − 14.70 and *R*^2^ = 0.94, respectively. However, it presented a less-fitted relationship between HR and difference in RMST estimated by IPCW regression with individual weights (*R*^2^ = 0.35 in univariable regression and *R*^2^ = 0.03 in multivariable regression). The decrease in *R*^2^ from 0.35 to 0.03 resulted from simultaneous inclusion of multiple covariates that violated assumption into multivariable IPCW regression with individual weights (Figure 2).

## 4. Discussion

When multiple covariates were of interest but violated PH assumption, time varying HRs were inconvenient to report. PH assumption-free RMST allowed reporting of effect measure for each covariate. Reporting results from both traditional survival methods and RMST-based methods could provide a comprehensive picture of effect. In RMST-based regressions, estimation from PV regression was more reliable and accurate than that from IPCW regression and had no limitation of homogeneity of censoring mechanism. In addition, including covariates that violate homogeneity of censoring mechanism assumption into IPCW regression would further reduce the accuracy of estimated RMST and statistical power. Robust and consistent negative logarithmic linear relationships between HRs and differences in RMST from PV regression supports the recommendation of PV regression and possible conversion between the two effect measures.

Clinical interpretation of HR may not be straightforward for clinicians [13]. If nonproportional hazards appear, a single HR value was biased because it was inconstant over time. RMST was supported to be an obligatory end point in oncological study [14]. RMST and difference (or ratio) in RMST showed several clinical and inferential advantages. Meanwhile, RMST could be converted to restricted mean survival lost, namely average of lost year from start to time point *τ*. RMST has been an appealing alternative to the HR. In a randomized controlled trial, to reflect the convenience of Internet-accessed sexually transmitted infection testing, difference in RMST was tested to assess difference in time to test [15]. A cohort study exploring the relationship between midlife cardiorespiratory fitness and chronic obstructive pulmonary disease performed both Cox models and RMST-based analysis, which increased the reliability and understandability of its conclusion [16]. In addition, RMST and difference in RMST had been considered as effect measures in meta-analysis [17, 18]. In RMST-based analysis, particular time *τ* specified would determine magnitude of difference or ratio in RMST and whether the difference or ratio is statistically significant or not. Fortunately, a step by step tutorial about study design, sample size estimation, and the determination of *τ* with RMST has been illustrated in details [19].

Trinquart et al. reconstructed individual patient data based on survival curves from 54 randomized controlled trials, and reestimated HRs, ratios of RMST, and differences in RMST [20]. In most trials, agreements regarding statistical significance of the three effect measures and direction of treatment effect were observed. However, the authors did not fit the relationship between HR and difference in RMST, and they were unable to validate the relationship among adjusted effect measures. In this study, a negative, well-fitted, and robust logarithmic relationship between HR and difference in RMST (unadjusted and adjusted) was observed for covariates meeting PH assumption. The relationship implied possibility of constructing function for conversion between difference in RMST and HR, which could play a vital role in meta-analysis.

Existence of confounders may distort true relationship between exposure and outcome, confounders-adjusted difference in RMST is essential. Furthermore, precision and statistical power could be improved after adjusting prognostic covariates when compared to RMST [21]. An approach developed by Zucker can estimate covariates-adjusted RMST effect based on Cox regression [22]. But, it was limited to calculate RMST effect of a binary variable of interest (such as experimental group and control group) adjusted for confounders. When the variable of interest has two or more two groups, standardized survival curves can be an alternative way [23]. This method has two steps: (1) defining a reference population for the confounders; (2) estimating adjusted curves for that population. The definition of reference population was a critical step which determines the generalization of adjusted survival curve. Two approaches had been proposed: marginal analysis (balance data on all confounders using reweighting before modeling) and conditional analysis (modeling a comprehensive overall model and getting average predicted curves from a series of predicted survival curves for any combination of confounders).

Both Zucker's method and standardized survival curves must make a clear division of covariates into variable of interest and confounders, and adjusted difference in RMST can be estimated only for categorical variable of interest. In addition, PH assumption was assumed to be hold as utilization of Cox regression in Zucker's method. Likewise, if Cox regression was adopted in modeling part of standardized survival curves, meeting PH assumption was also required. Complex programming and difficulty in extension limited their friendly applications. RMST-based regressions, using generalized linear modeling technique, can study effects of single factors or multiple factor on RMST without limitation on variable type and distinction between key variable and confounding variables. Model-based inference and prediction can be practiced. Moreover, varied regression-based methods were allowed, such as nonlinear fitting and interaction effect.

In IPCW regression, weights estimation was the key step based on a correctly specified censoring distribution. When a categorical covariate of interest presented, individual weights or group-specified weights should be determined based on result of testing on homogeneity of censoring mechanism assumption [24]. IPCW regression with group-specified weights was the correct selection when the covariate broke the assumption. When a categorical covariate satisfied the assumption, parameters of censoring distribution in each subgroup were similar to those in a complete data. Meanwhile, a group-specified weight could also be used to obtain accurate estimation. In this study, group-specified weights gave more accurate estimates than individual weights for both categorical covariates that met assumption and those that did not meet. However, in a multivariable regression, if more than one categorical covariates of interest violated the assumption, it was hard to specify multiple stratums when performing IPCW with group-specific weights. In this study, forced adoption of IPCW with individual weights presented biased and misleading difference in RMST. In addition, inverse probability might create extreme weight when the probability is close to 0, and small sample size might make inverse probability instable [25]. With such restrictions on IPCW regression, PV regression should be a preferred method when exploring effects of multiple covariates of interest. Codes for pseudovalue method have been developed in three main platforms (SAS, R, and Stata) [12, 26, 27].

### 4.1. A Comprehensive Flowchart for Application

**Figure 3:**
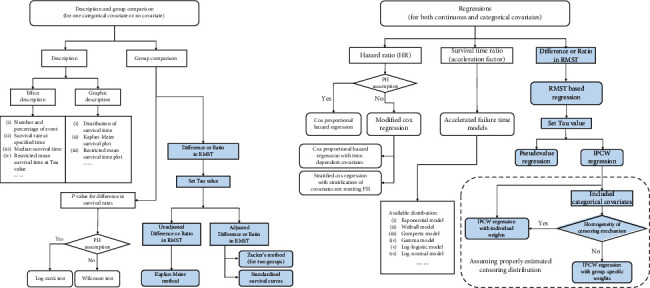
A flowchart combined traditional survival methods and restricted mean survival time based methods.

To comprehensively present these methods, we drew a flowchart with two parts, (1) description and group comparison; (2) regressions (see Figure 3). RMST-based methods were presented with blue background.

In part 1, statistical description for survival data includes summary measures (such as number of event, median survival time, and RMST) and graphic description (such as KM curves). When a categorical covariate is of interest, group comparison should be performed for difference in survival rates and RMST. When you want to estimate difference or ratio in RMST between groups, a truncation time *τ* need to be prespecified. Unadjusted difference or ratio in RSMT can be estimated by KM curve directly, adjusted one can be estimated by standardized KM curve or Zucker's method. In part 2, both categorical and continuous covariates are allowed. Unadjusted and adjusted HR, survival time ratio (acceleration factor) and difference (or ratio) in RMST can be estimated as target effect measures. In general, HR greater than 1 implies that exposure is harmful to survival, acceleration factor and ratio in RMST implies that exposure benefits survival. If PH assumption is not satisfied, HR can be estimated by Cox regression with time-dependent covariates or stratified Cox regression with different baseline hazard rate in each stratification. Ratio of survival times (acceleration factor) can be estimated in acceleration failure time (AFT) models, which require to prespecify distribution of survival time and check AFT assumption, such as Weibull distribution. Lastly, PV regression and IPCW regressions are used to assess difference (or ratio) in RMST, and the *τ* need to be prespecified.

### 4.2. Limitation

This study had some limitations. Firstly, *τ* specified in RMST-based analysis determined the magnitude of difference in RMST and whether the difference was statistically significant or not, a general strategy to select *τ* was applied but influence of changeable *τ* was not studied. Secondly, the estimated coefficients in nonlinear relationship between HR and difference in RMST was not generalized for conversion in other study, a general conversion function was expected to be developed. Lastly, not all survival analyses for right-censored data were integrated into the flowchart, survival analyses on left-censored data, interval-censored data, recurrent event ,and competing risk were not discussed.

## 5. Conclusion

HR and difference in RMST should be reported with equal consideration to present effects comprehensively and to improve the communication of clinical evidence. When more than one covariates were of interest, KM method and PV regression could be the preferred ones in RMST-based methods. The flowchart that incorporates traditional survival methods and RMST-based methods can help clinicians and layperson to select appropriate methods.

## Figures and Tables

**Table 1:** Assessment for proportional hazard assumption and homogeneity of censoring mechanism assumption.

Covariates	Proportional hazard assumption		Homogeneity of censoring mechanism	
	KM curves	Ln (-ln(S(t))) vs. ln(t) curves	Graphical analysis	Log-rank test
Age (continuous)	—	—	—	—
Age (binary)	✓	✓	✓	✓
Sex	×	×	✓	✓
Year of diagnosis	✓	✓	×	×
Race	×	×	✓	✓
Tumor size	×	×	×	×
Extend of disease	✓	✓	×	×
AJCC	✓	×	×	×
Surgery	✓	✓	×	×
Radiation	✓	✓	✓	✓
Chemotherapy	×	×	×	×

KM, Kaplan-Meier. AJCC, American Joint Committee on Cancer for staging.

**Table 2:** Basic characteristics and univariable analyses including traditional methods and restricted mean survival time based methods.

Covariates	Description	Group comparison			Univariable regression			
		Survival rate		Difference in RMST ^a^	Cox regression ^b^	RMST-based regression		
		Log-rank	Wilcoxon			PV regression ^a^	IPCW regression (individual weights) ^a^	IPCW regression (group-specified weight) ^a^
Age (continuous)	30 (19, 54)	—	—	—	1.13, <0.001	-4.09, <0.001	-4.35, <0.001	—
Age (binary)								
>60 years	841(19.09%)	<0.001	<0.001	-146.64, <0.001	3.82, <0.001	-147.29, <0.001	-137.17, <0.001	-146.43, <0.001
< =60 years	3564(80.91%)	—	—	Reference	Reference	Reference	Reference	Reference
Sex								
Male	2513(57.05%)	0.005	0.058	-24.31, 0.004	Time-varying	-24.22, 0.004	-60.12, 0.46	-23.98, 0.42
Female		—	—	Reference		Reference	Reference	Reference
Year of diagnosis								
1970s~1980s	803 (18.23%)	<0.001	<0.001	-26.89, <0.001	1.32, <0.001	-28.03, <0.001	178.96, <0.001	-26.27, <0.001
1990s~2000s	3602(81.77%)	—	—	Reference	Reference	Reference	Reference	Reference
Race								
White	3373(76.57%)	0.04	0.004	-12.60, 0.21	Time-varying	-12.15, 0.21	-40.21, 0.71	-12.80, 0.74
Others	1032(23.43%)	—	—	Reference		Reference	Reference	Reference
Tumor size		<0.001^∗^	<0.001^∗^	<0.001				
>100 mm	209(4.74%)	—	—	-50.03, <0.001	Time-varying	-58.56, <0.001	-33.62, 0.010	-50.02, 0.07
Unknown	3541(80.39%)	—	—	-36.45, <0.001	Time-varying	-39.35, <0.001	72.64, <0.001	-37.02, <0.001
<100 mm	655(14.87%)	—	—	—			Reference	Reference
Extend of disease		<0.001^∗^	<0.001^∗^	<0.001^∗^				
Metastasis	887(20.14%)	—	—	-164.09, <0.001	6.34, <0.001	-183.43, <0.001	-113.45, <0.001	-164.05, <0.001
Local invasion	261(5.93%)	—	—	-60.15, <0.001	1.98, <0.001	-71.54, <0.001	-22.99, <0.001	-60.18, <0.001
Unknown	2974(67.51%)	—	—	-82.28, <0.001	2.50, <0.001	-93.62, <0.001	2.21, <0.001	-82.94, <0.001
Confined	283(6.42%)	—	—	Reference	Reference	Reference	Reference	Reference
AJCC		<0.001^∗^	<0.001^∗^	<0.001^∗^				
III + IV	421(9.56%)	—	—	-84.75, <0.001	Time-varying	-74,77, <0.001	-67.47, <0.001	-84.90, <0.001
II	886(20.11%)	—	—	-33.62, <0.001	Time-varying	-26.51, <0.001	-41.22, <0.001	-33.82, <0.001
Unknown	2891(65.63%)	—	—	-45.51, <0.001	Time-varying	-38.16, <0.001	-3.95, 0.11	-45.40, <0.001
I	207(4.07%)	—	—	Reference		Reference	Reference	Reference
Surgery, yes	3427(77.80%)	<0.001	<0.001	121.41, <0.001	0.37, <0.001	121.11, <0.001	18.17, 0.88	121.05, <0.001
Radiation, yes	621(14.10%)	<0.001	<0.001	-133.53, <0.001	2.54, <0.001	-134.41, <0.001	-29.15, 0.73	-133.39, <0.001
Chemotherapy, yes	2959(67.17%)	<0.001	<0.001	16.26, 0.048	Time-varying	17.04, 0.05	-137.623, 0.001	16.06, 0.27

RMST, restricted mean survival time. IPCW, inverse probability of censored weighting. AJCC, American Joint Committee on Cancer for staging. ^a^ Presented as point estimation of coefficient (difference in RMST) and corresponding *p* value. ^b^ Presented as point estimation of hazard ratio and corresponding *p* value or “time-varying”. ^∗^. Overall test for categorical covariates with more than 2 groups.

**Table 3:** Multivariable analysis based on cox proportional hazard regression and restricted mean survival time based regression.

Parameter	Cox regression		Pseudovalue regression		IPCW regression (individual weights)	
	HR (95% CI)	*p*	Difference in RSMT (95% CI)	*p*	Difference in RSMT (95% CI)	*p*
Age (continuous)	1.03(1.03,1.03)	<0.001	-3.89(-4.22,-3.55)	<0.001	-2.65(-3.18,-2.11)	<0.001
Sex, male	Time-varying		-24.85(-38.64,-11.06)	<0.001	-18.38(-46.87,10.11)	0.206
Year of diagnosis						
1970s~1980s	1.41(1.25,1.59)	<0.001	-28.38(-2.68,-54.07)	0.03	-195.11(-224.25,-165.96)	<0.001
1990s~2000s	Reference		Reference		Reference	
Race						
White and black	Time-varying		-10.73(-26.64,5.19)	0.186	-27.61(-52.66,-2.55)	0.031
Other	Reference		Reference		Reference	
Tumor size						
>100 mm	Time-varying		-49.38(-84.87,-13.89)	0.006	-15.53(-30.21,-0.85)	0.038
Unknown	Time-varying		-11.82(-38.20,14.55)	0.38	-5.90(-20.14,8.35)	0.417
<100 mm	Reference		Reference		Reference	
EOD						
Metastasis	4.44(3.46,5.69)	<0.001	-202.21(-237.34,-167.08)	<0.001	-20.45(-43.36,2.46)	0.08
Local invasion	1.65(1.32,2.05)	<0.001	-89.90(-121.74,-58.06)	<0.001	1.68(-12.77,16.13)	0.82
Unknown	1.79(1.41,2.27)	<0.001	-113.04(-147.17,-78.91)	<0.001	38.85(23.92,53.78)	<0.001
Confined	Reference		Reference		Reference	
AJCC staging						
III + IV	Time-varying		-168.85(-196.05,-141.65)	<0.001	25.66(-4.16,55.47)	0.092
II	Time-varying		-61.40(-86.12,-36.68)	<0.001	19.42(-4.98,43.81)	0.119
Unknown	Time-varying		-88.88(-114.01,-63.75)	<0.001	73.84(54.56,93.12)	<0.001
I	Reference		Reference		Reference	
Surgery, yes	0.58(0.53,0.64)	<0.001	36.22(19.23,53.21)	<0.001	53.01(17.35,88.67)	0.004
Radiation, yes	1.44(1.30,1.60)	<0.001	-45.76(-63.27,-28.25)	<0.001	-6.52(-58.84,45.80)	0.807
Chemotherapy, yes	Time-varying		-49.93(-67.87,-31.99)	<0.001	-58.58(-97.25,-19.91)	0.003

RMST, restricted mean survival time. IPCW, inverse probability of censored weighting. AJCC, American Joint Committee on Cancer for staging.

## Data Availability

Data and SAS code can be available upon request from all authors.

## Abbreviations

HR: Hazard ratio
IPCW: Inverse probability of censoring probability
pH: Proportional hazard
PV: Pseudovalue
RMST: Restricted mean survival time
SEER: The surveillance, epidemiology, and end results
AJCC: American joint committee on cancer for staging
STG: Survival time gain
STL: Survival time lost.

## Supplementary Materials

Supplementary Materials Graphic assessment of proportional hazard assumption: graphic assessments (Kaplan-Meier curves and ln (-ln(S(t))) vs. ln(t) Curves) of proportional hazard assumption for included covariates.

## References

[1] Clark T. G., Bradburn M. J., Love S. B., Altman D. G. (2003). Survival analysis part I: basic concepts and first analyses. *British Journal of Cancer*.

[2] Bradburn M. J., Clark T. G., Love S. B., Altman D. G. (2003). Survival analysis part II: multivariate data analysis – an introduction to concepts and methods. *British Journal of Cancer*.

[3] Blagoev K. B., Wilkerson J., Fojo T. (2012). Hazard ratios in cancer clinical trials—a primer. *Nature Reviews Clinical Oncology*.

[4] Hernan M. A. (2010). The hazards of hazard ratios. *Epidemiology*.

[5] Royston P., Parmar M. K. B. (2011). The use of restricted mean survival time to estimate the treatment effect in randomized clinical trials when the proportional hazards assumption is in doubt. *Statistics in Medicine*.

[6] Royston P., Parmar M. K. (2013). Restricted mean survival time: an alternative to the hazard ratio for the design and analysis of randomized trials with a time-to-event outcome. *BMC Medical Research Methodology*.

[7] Vandenbroucke J. P., von Elm E., Altman D. G. (2014). Strengthening the reporting of observational studies in epidemiology (STROBE): explanation and elaboration. *International Journal of Surgery*.

[8] Schulz K. F., Altman D. G., Moher D., for the CONSORT Group (2010). CONSORT 2010 statement: updated guidelines for reporting parallel group randomised trials. *BMJ*.

[9] Tian L., Zhao L., Wei L. J. (2014). Predicting the restricted mean event time with the subject's baseline covariates in survival analysis. *Biostatistics*.

[10] Andersen P. K., Hansen M. G., Klein J. P. (2004). Regression analysis of restricted mean survival time based on pseudo-observations. *Lifetime Data Analysis*.

[11] Bellera C. A., MacGrogan G., Debled M., de Lara C. T., Brouste V., Mathoulin-Pélissier S. (2010). Variables with time-varying effects and the cox model: some statistical concepts illustrated with a prognostic factor study in breast cancer. *BMC Medical Research Methodology*.

[12] Klein J. P., Gerster M., Andersen P. K., Tarima S., Perme M. P. (2008). SAS and R functions to compute pseudo-values for censored data regression. *Computer Methods and Programs in Biomedicine*.

[13] Calkins K. L., Canan C. E., Moore R. D., Lesko C. R., Lau B. (2018). An application of restricted mean survival time in a competing risks setting: comparing time to ART initiation by injection drug use. *BMC Medical Research Methodology*.

[14] A’Hern R. P. (2016). Restricted mean survival time: an obligatory end point for time-to-event analysis in cancer trials?. *Journal of Clinical Oncology*.

[15] Wilson E., Free C., Morris T. P. (2017). Internet-accessed sexually transmitted infection (e-STI) testing and results service: a randomised, single-blind, controlled trial. *PLoS Medicine*.

[16] Hansen G. M., Marott J. L., Holtermann A., Gyntelberg F., Lange P., Jensen M. T. (2019). Midlife cardiorespiratory fitness and the long-term risk of chronic obstructive pulmonary disease. *Thorax*.

[17] Niglio S. A., Jia R., Ji J. (2019). Programmed death-1 or programmed death ligand-1 blockade in patients with platinum-resistant metastatic urothelial cancer: a systematic review and meta-analysis. *European Urology*.

[18] Wei Y., Royston P., Tierney J. F., Parmar M. K. (2015). Meta-analysis of time-to-event outcomes from randomized trials using restricted mean survival time: application to individual participant data. *Statistics in Medicine*.

[19] Pak K., Uno H., Kim D. H. (2017). Interpretability of cancer clinical trial results using restricted mean survival time as an alternative to the hazard ratio. *JAMA Oncology*.

[20] Trinquart L., Jacot J., Conner S. C., Porcher R. (2016). Comparison of treatment effects measured by the hazard ratio and by the ratio of restricted mean survival times in oncology randomized controlled trials. *Journal of Clinical Oncology*.

[21] Karrison T., Kocherginsky M. (2018). Restricted mean survival time: does covariate adjustment improve precision in randomized clinical trials?. *Clinical Trials*.

[22] Zucker D. M. (1998). Restricted mean life with covariates: modification and extension of a useful survival analysis method. *Journal of the American Statistical Association*.

[23] Therneau T. M., Grambsch P. M. (2000). *Modeling Survival Data: Extending the Cox Model*.

[24] Willems S., Schat A., van Noorden M. S., Fiocco M. (2018). Correcting for dependent censoring in routine outcome monitoring data by applying the inverse probability censoring weighted estimator. *Statistical Methods in Medical Research*.

[25] Robins J., Sued M., Lei-Gomez Q., Rotnitzky A. (2007). Comment: performance of double-robust estimators when “inverse probability” weights are highly variable. *Statistical Science*.

[26] Parner E. T., Andersen P. K. (2010). Regression analysis of censored data using pseudo-observations. *The Stata Journal*.

[27] Qiao H., Jun L., Bing-Hui L., Lin-Lu M., Tong D., Xian-Tao Z. (2019). A univariable and multivariable analysis of right censored time-to-event data based on restricted mean survival time: a combination with traditional survival methods. *Research Square*.

