# Supplementary material for: Performance of Restricted Mean Survival Time Based Methods and Traditional Survival Methods: An Application in an Oncological Data
Source: Comput Math Methods Med. 2022 Dec 28;2022:7264382. doi: 10.1155/2022/7264382 (PMC9812622; doi:10.1155/2022/7264382)

Check Proportional hazard assumption

(KM curves and ln (-ln(S(t))) vs ln(t) Curves)

Dichotomous age

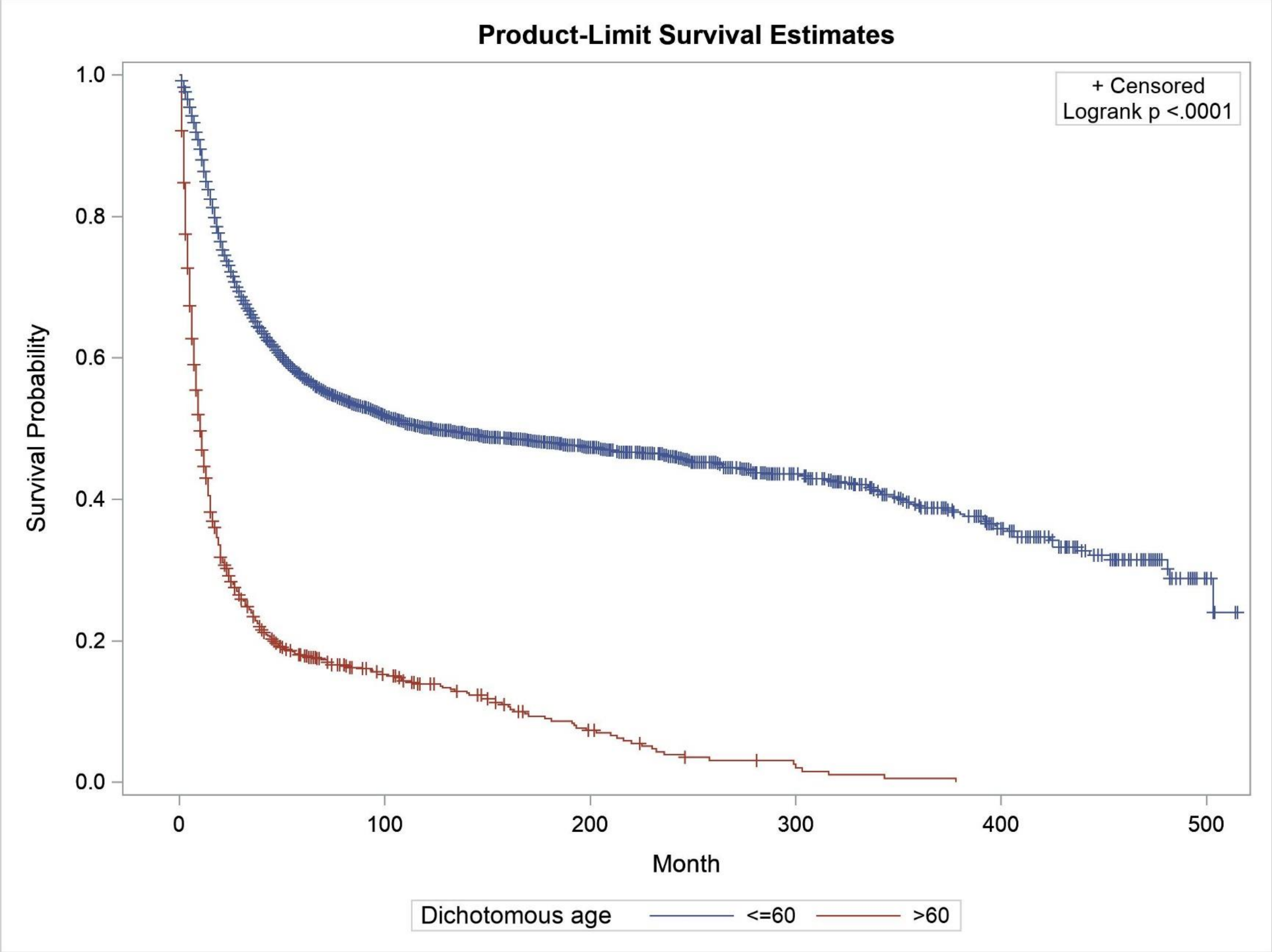

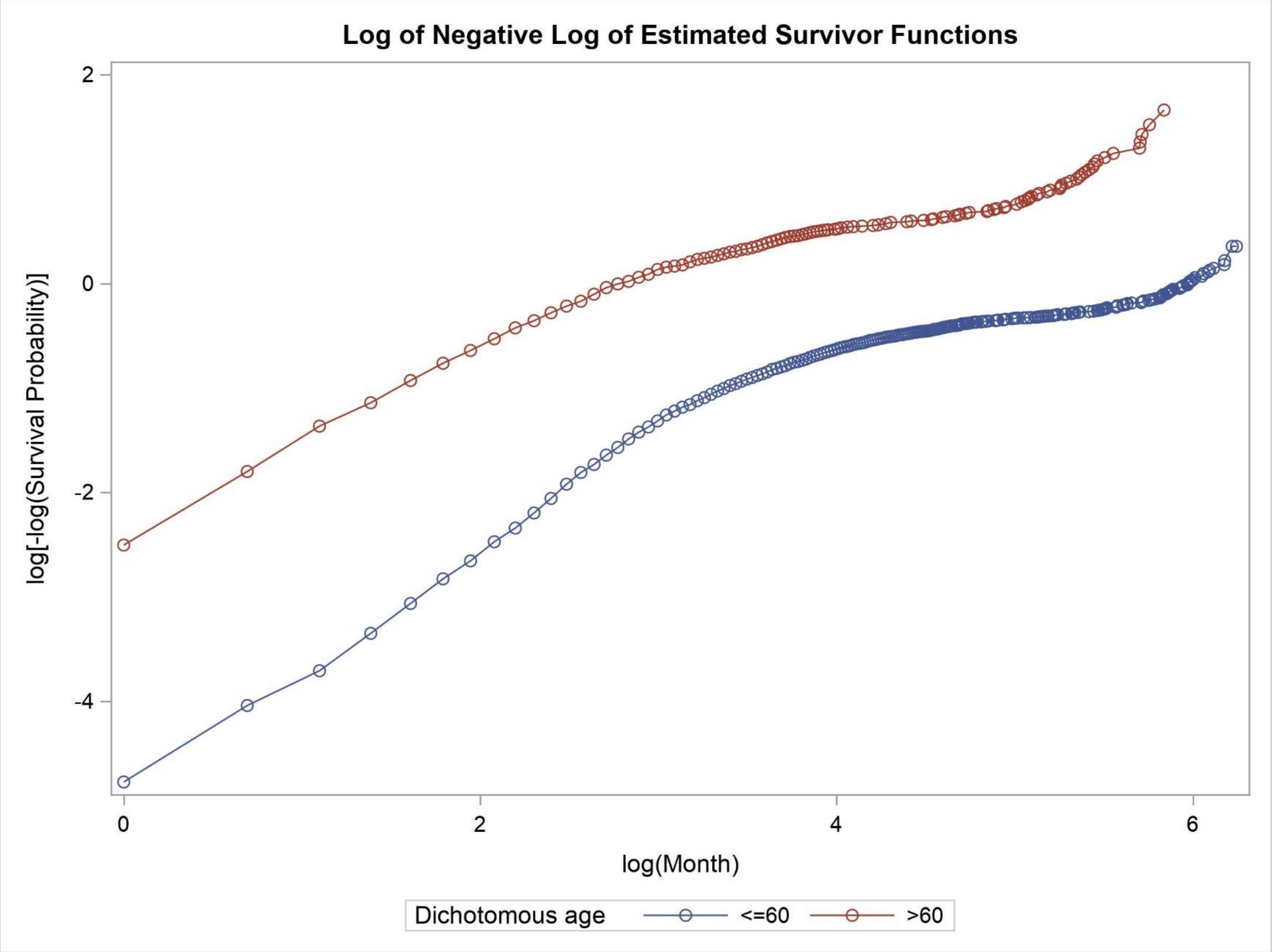

Sex

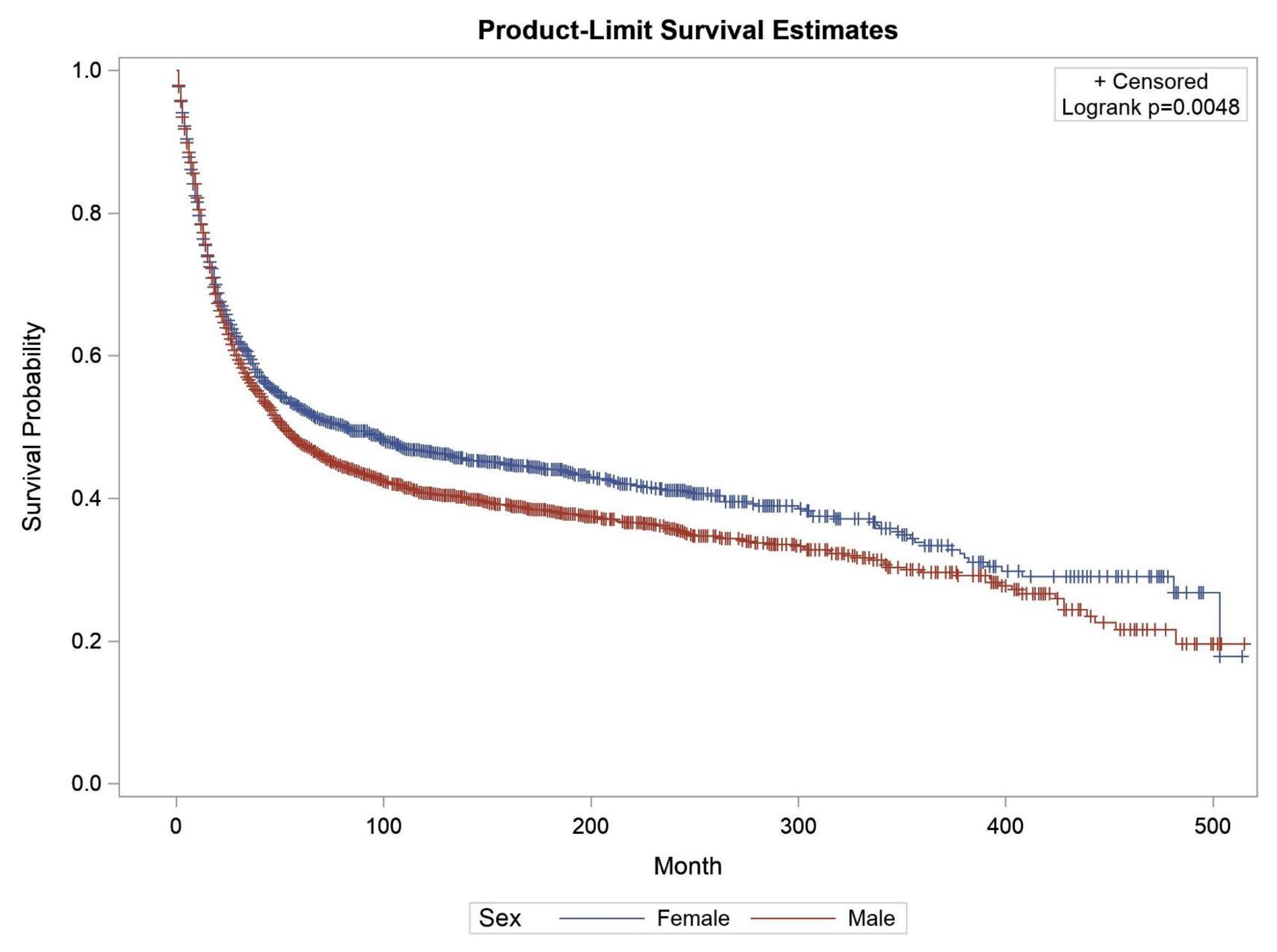
Product-Limit Survival Estimates
+ Censored
Logrank p=0.0048
1.0
0.8
0.6
0.4
0.2
0.0
Survival Probability
0
100
200
300
400
500
Month
Sex
Female
Male

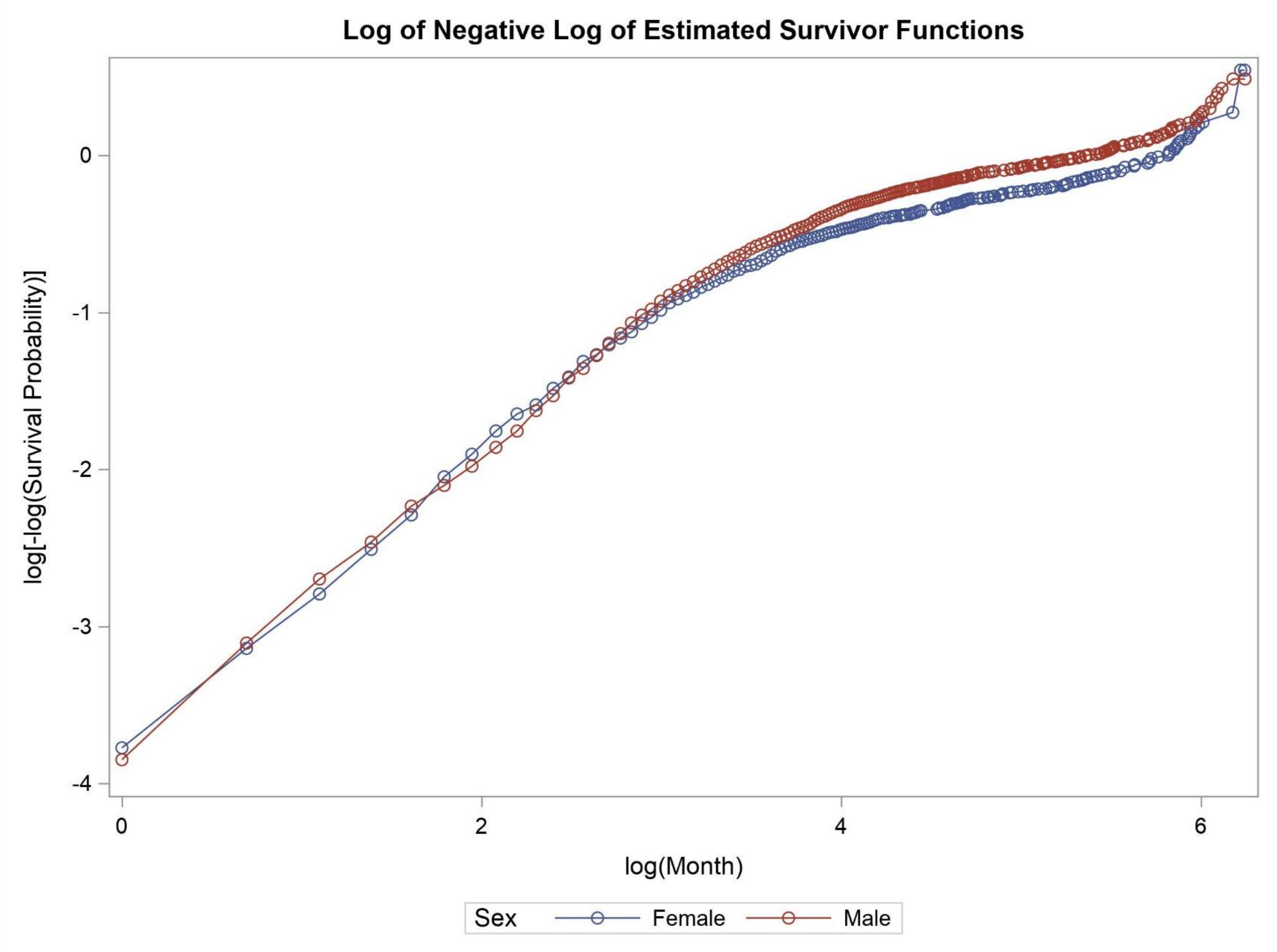
Log of Negative Log of Estimated Survivor Functions
0
-1
-2
-3
-4
log[-log(Survival Probability)]
0
2
4
6
log(Month)
Sex
Female
Male

Year of diagnosis

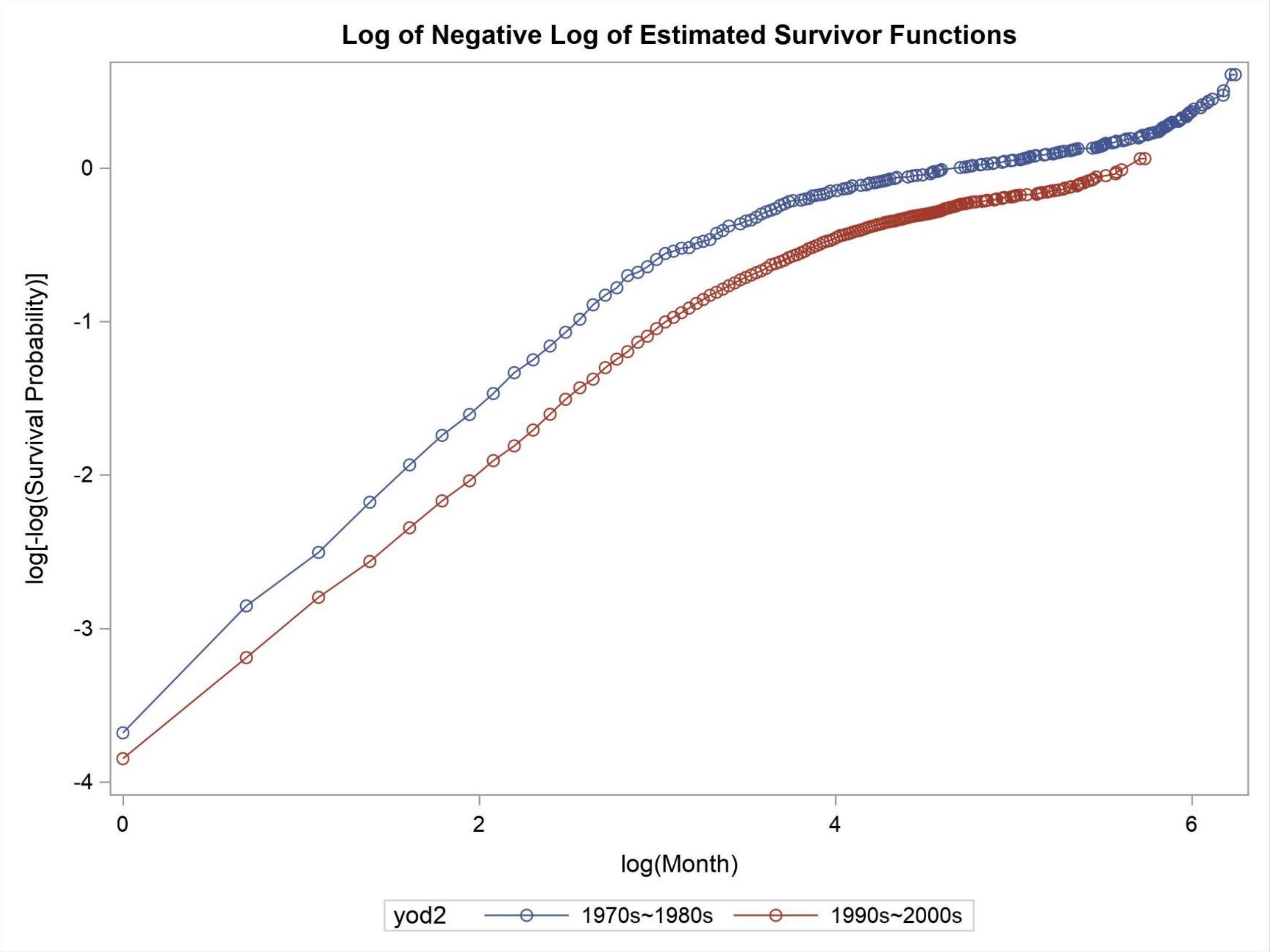

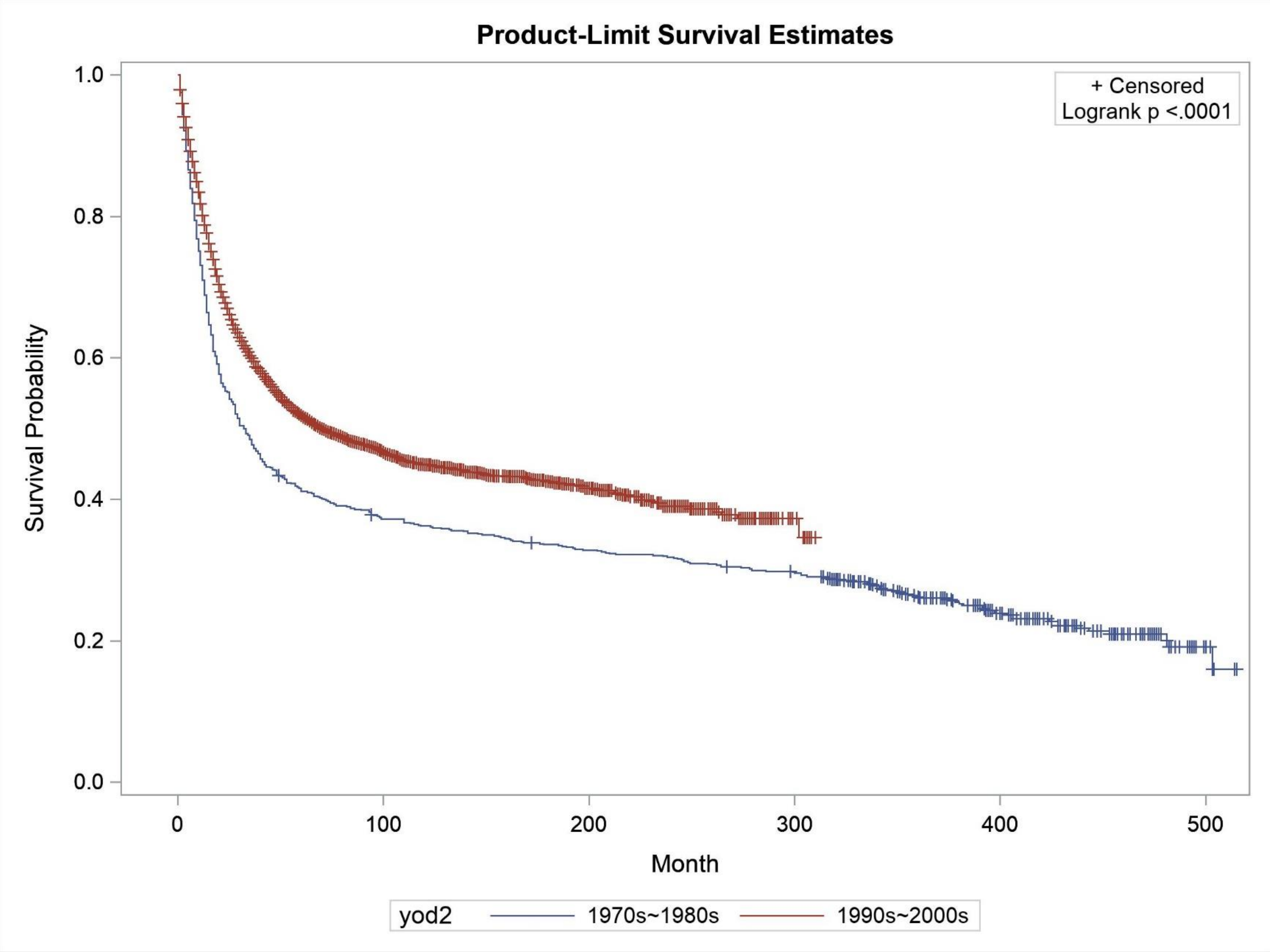

Race

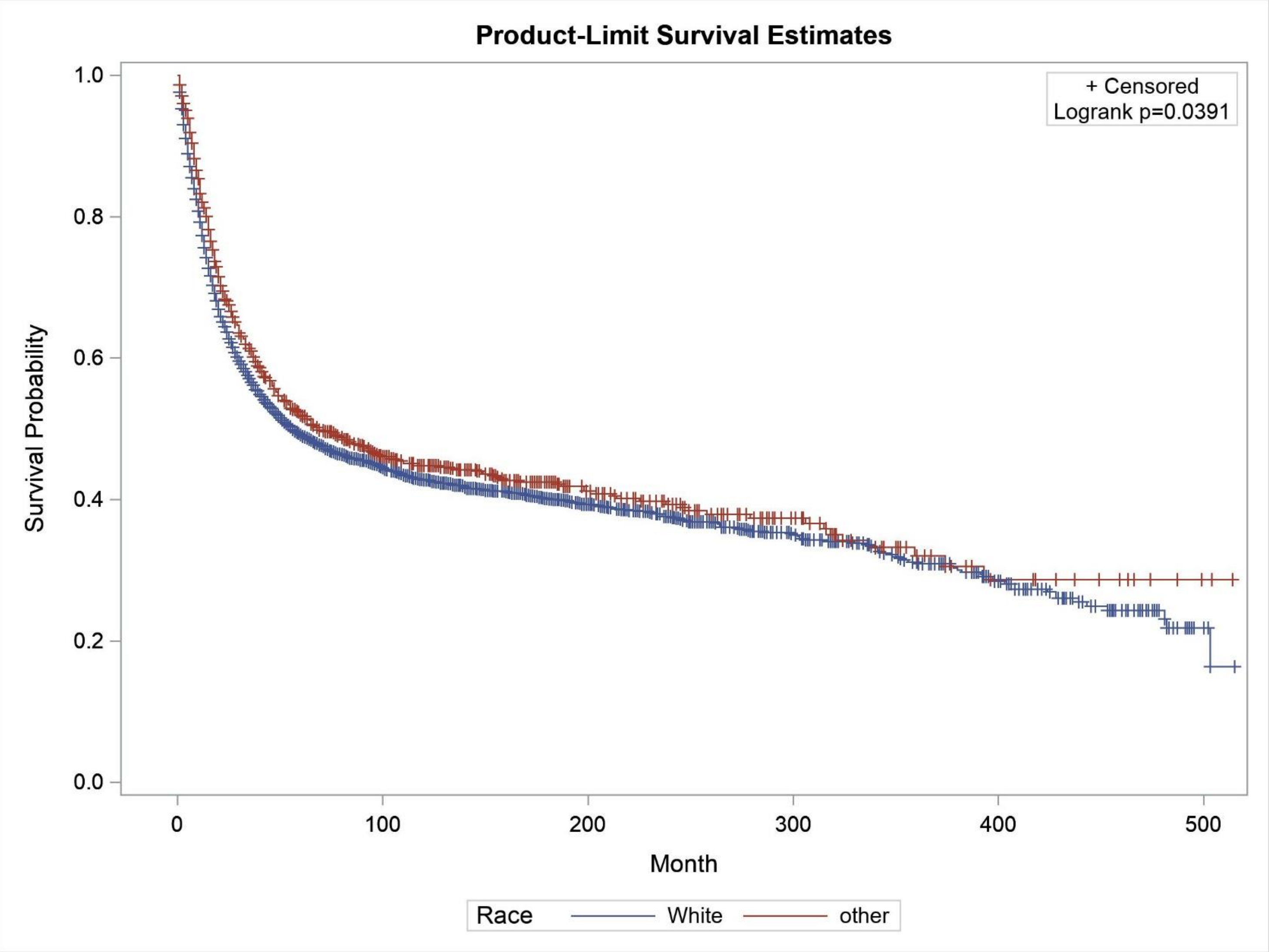

Product-Limit Survival Estimates
+ Censored
Logrank p=0.0391
Survival Probability
1.0
0.8
0.6
0.4
0.2
0.0
0
100
200
300
400
500
Month
Race
White
other

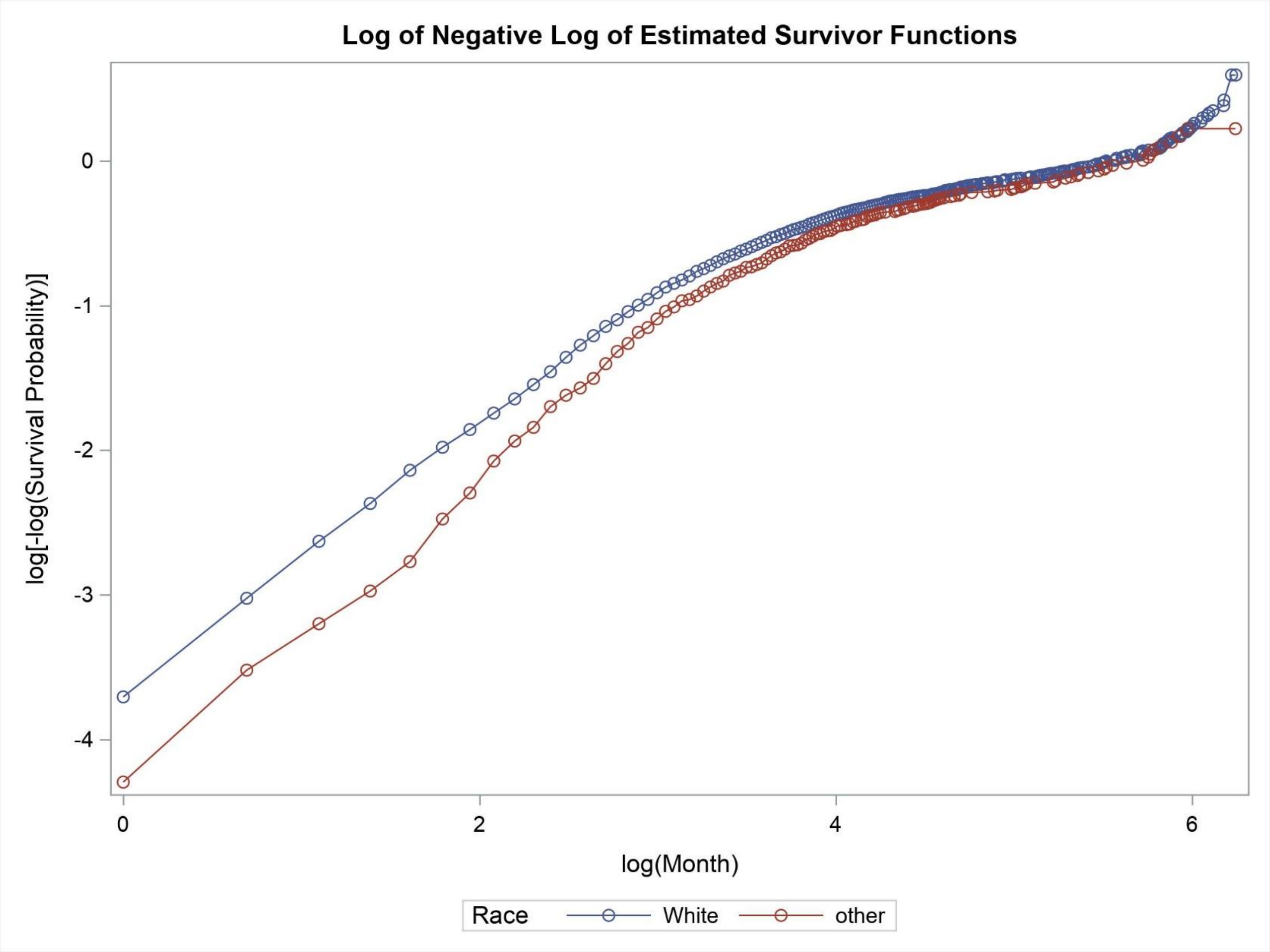

Log of Negative Log of Estimated Survivor Functions
log[-log(Survival Probability)]
0
-1
-2
-3
-4
0
2
4
6
log(Month)
Race
White
other

Tumor size

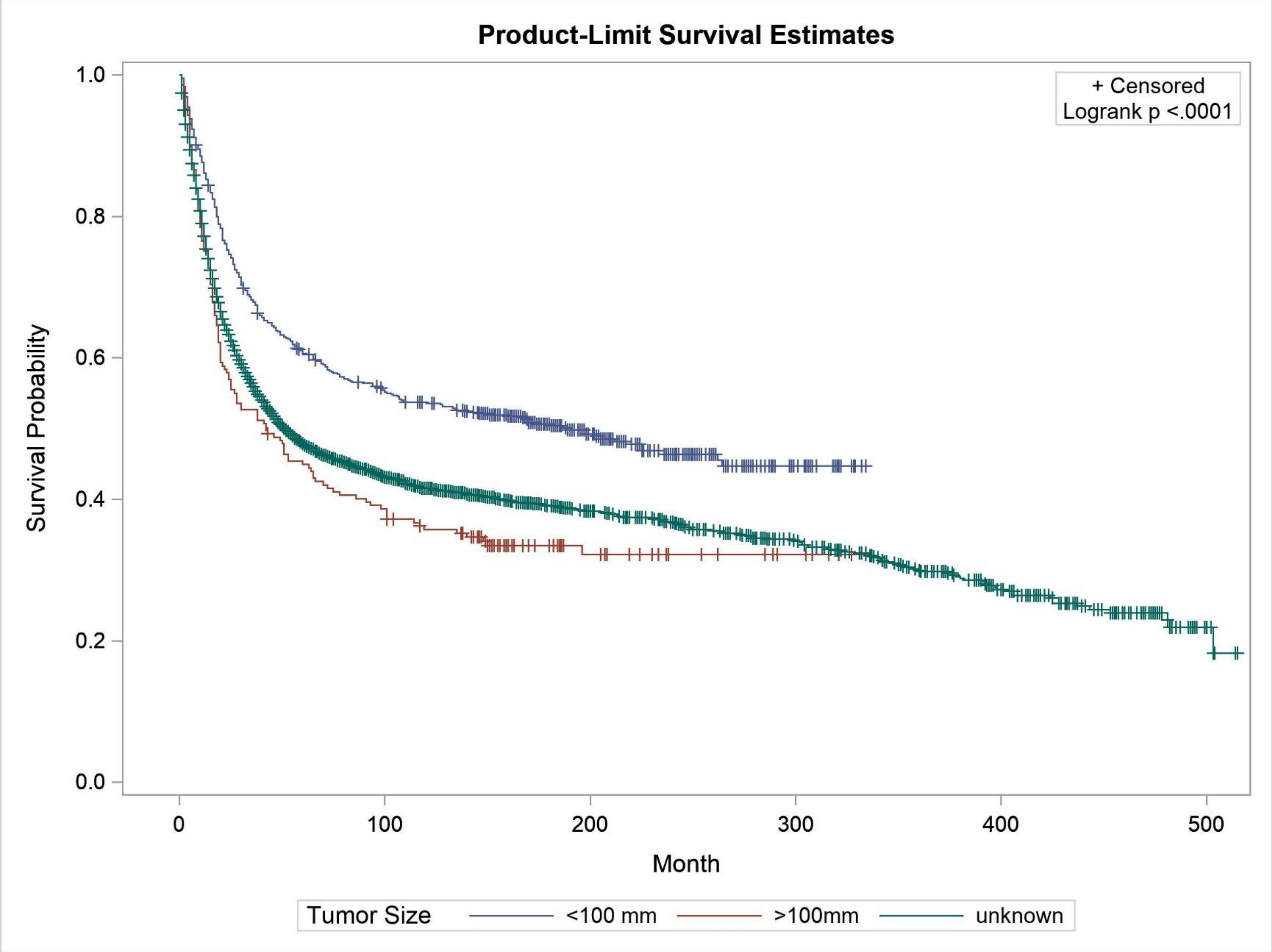
Product-Limit Survival Estimates
+ Censored
Logrank p <.0001
Survival Probability
1.0
0.8
0.6
0.4
0.2
0.0
0
100
200
300
400
500
Month
Tumor Size
<100 mm
>100mm
unknown

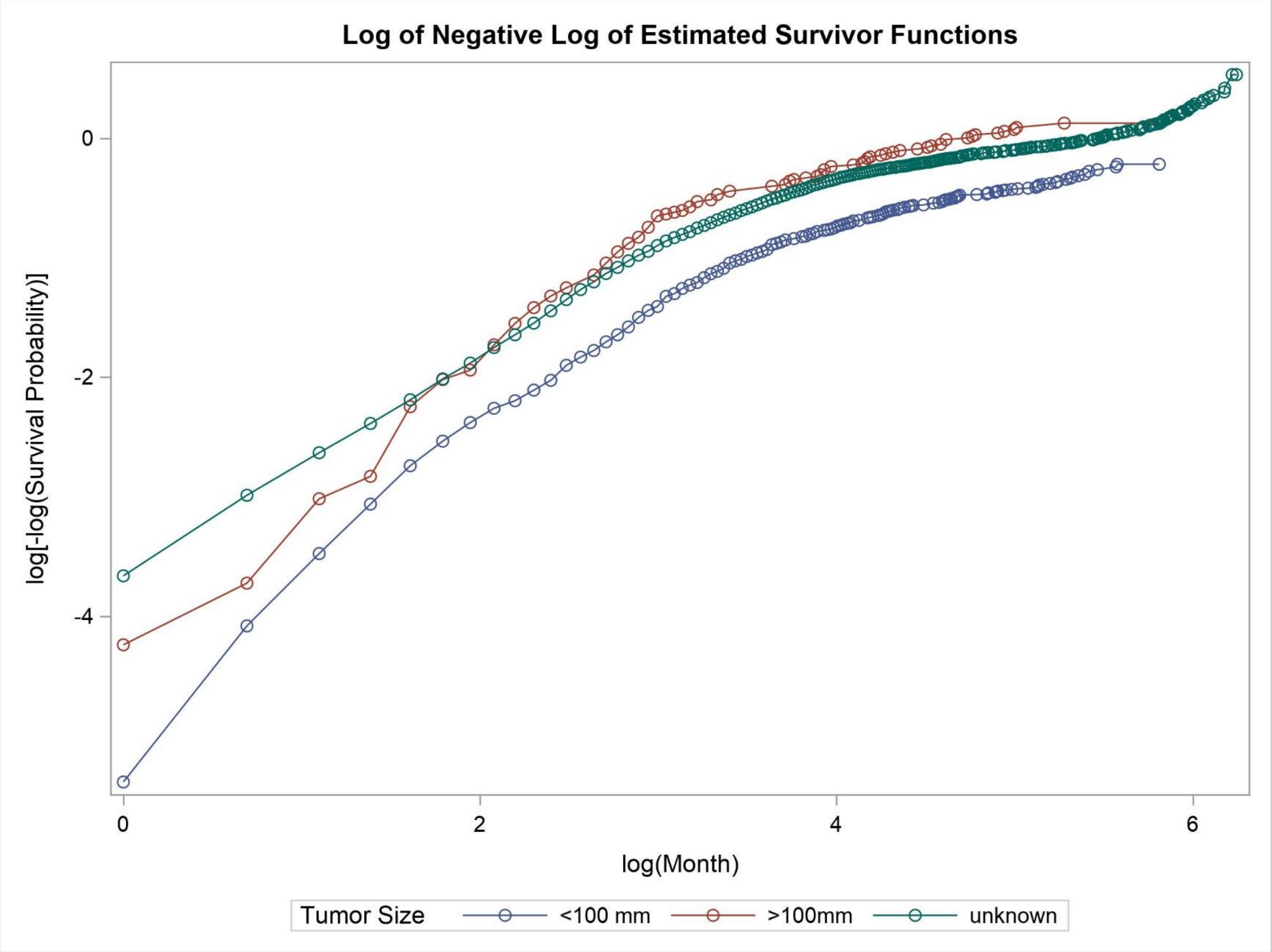
Log of Negative Log of Estimated Survivor Functions
log[-log(Survival Probability)]
0
-2
-4
0
2
4
6
log(Month)
Tumor Size
<100 mm
>100mm
unknown

Extend of disease

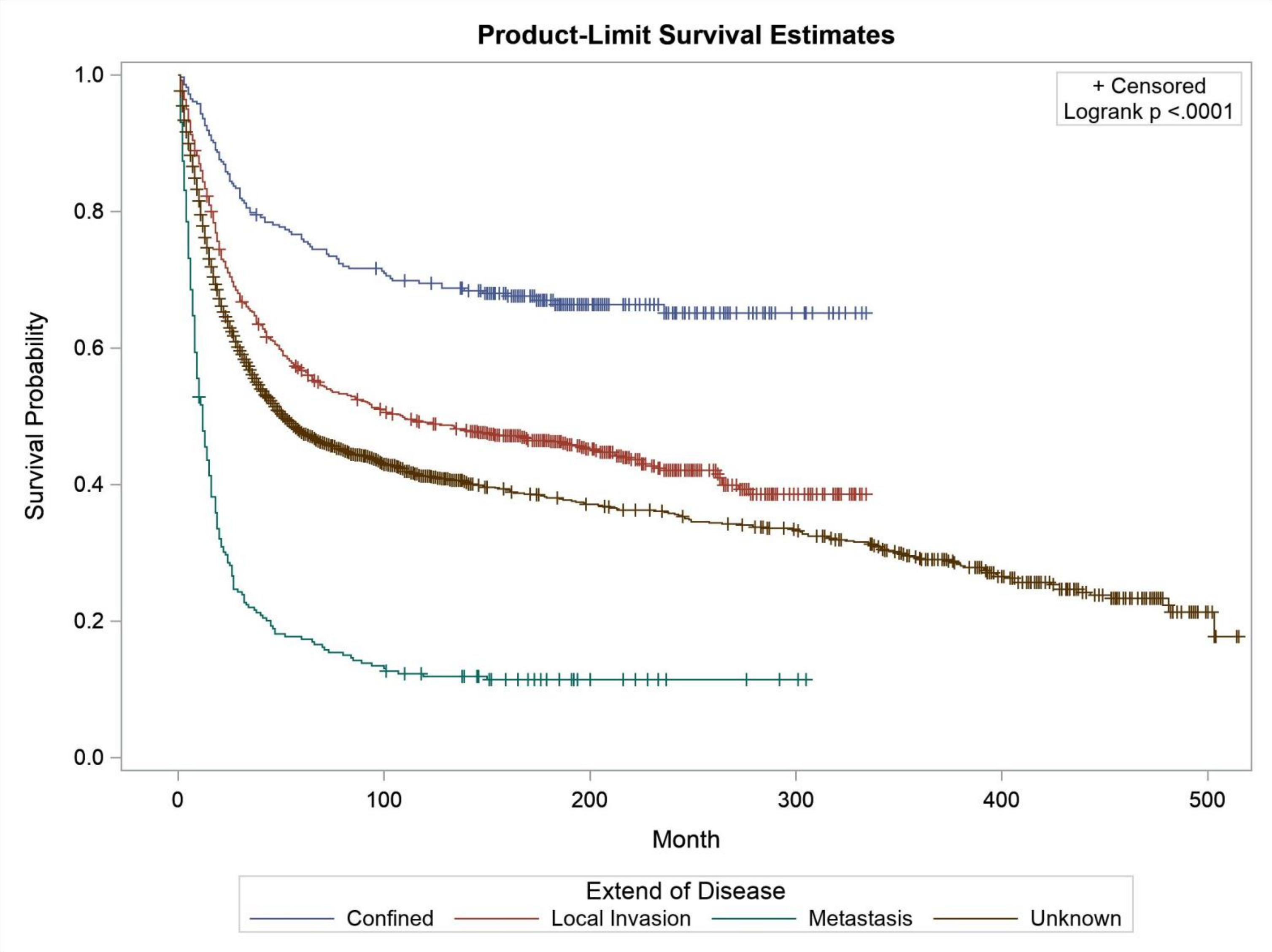

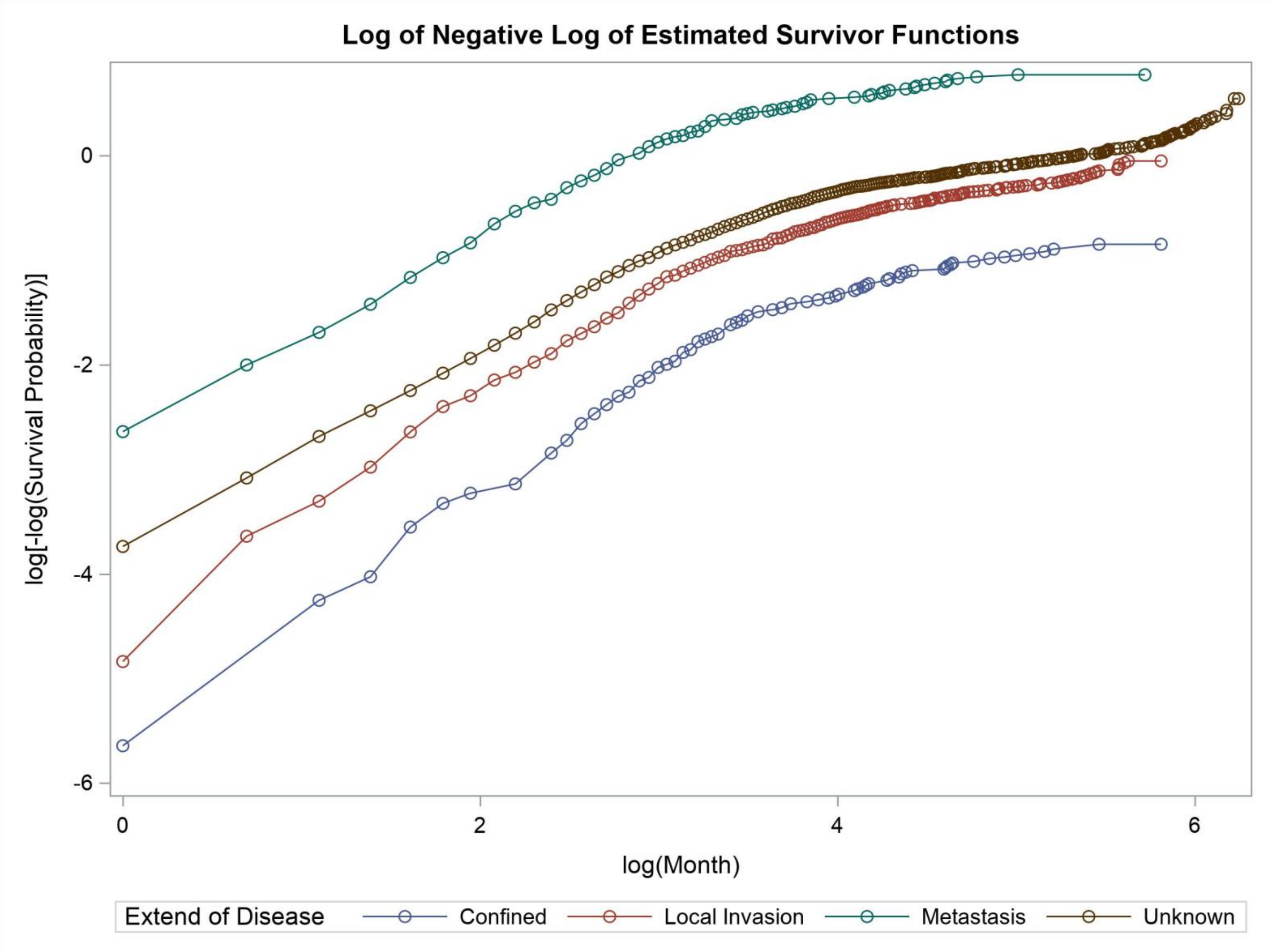

AJCC stage

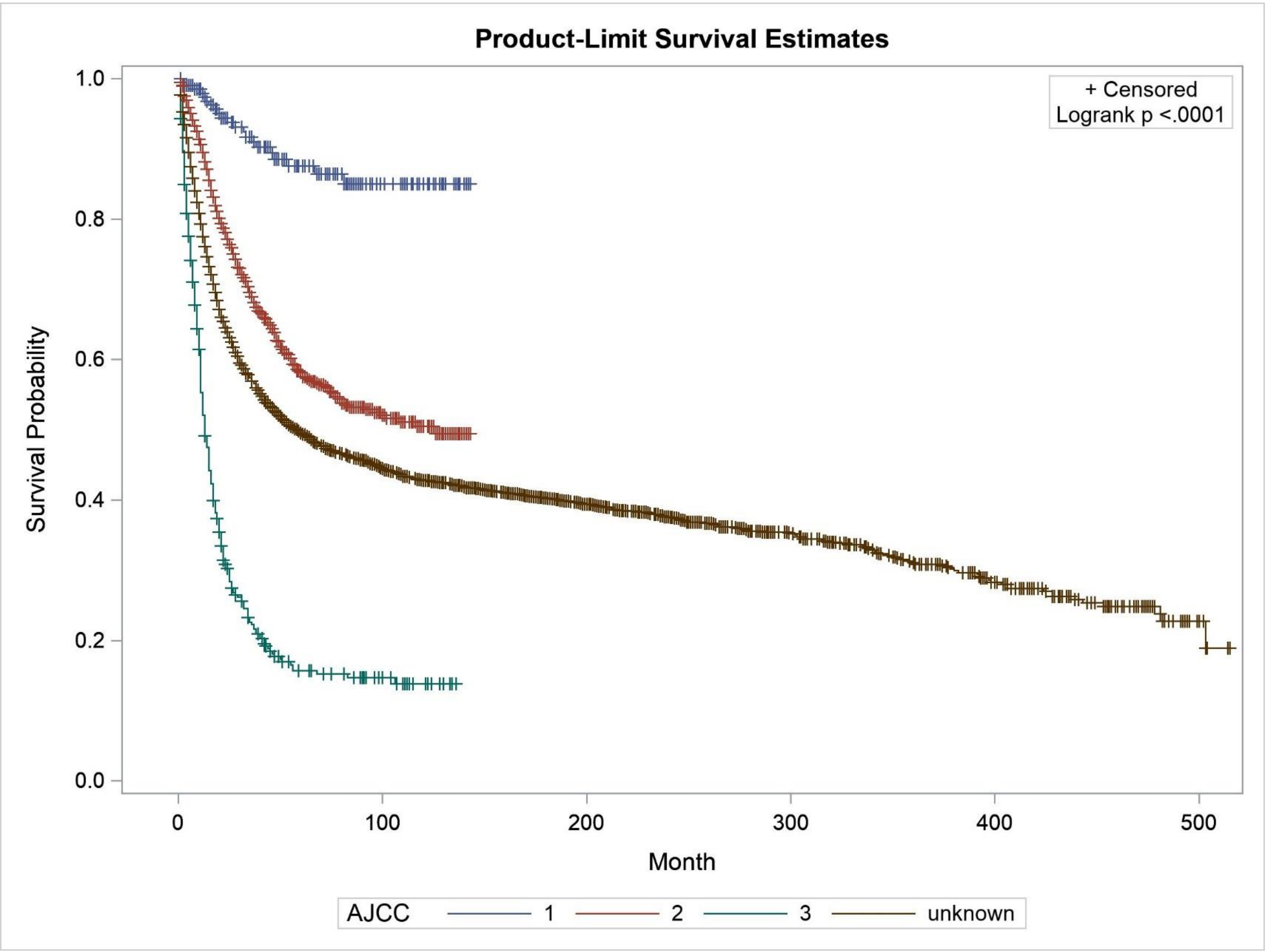

Product-Limit Survival Estimates
+ Censored
Logrank p <.0001
Survival Probability
1.0
0.8
0.6
0.4
0.2
0.0
0
100
200
300
400
500
Month
AJCC 1 2 3 unknown

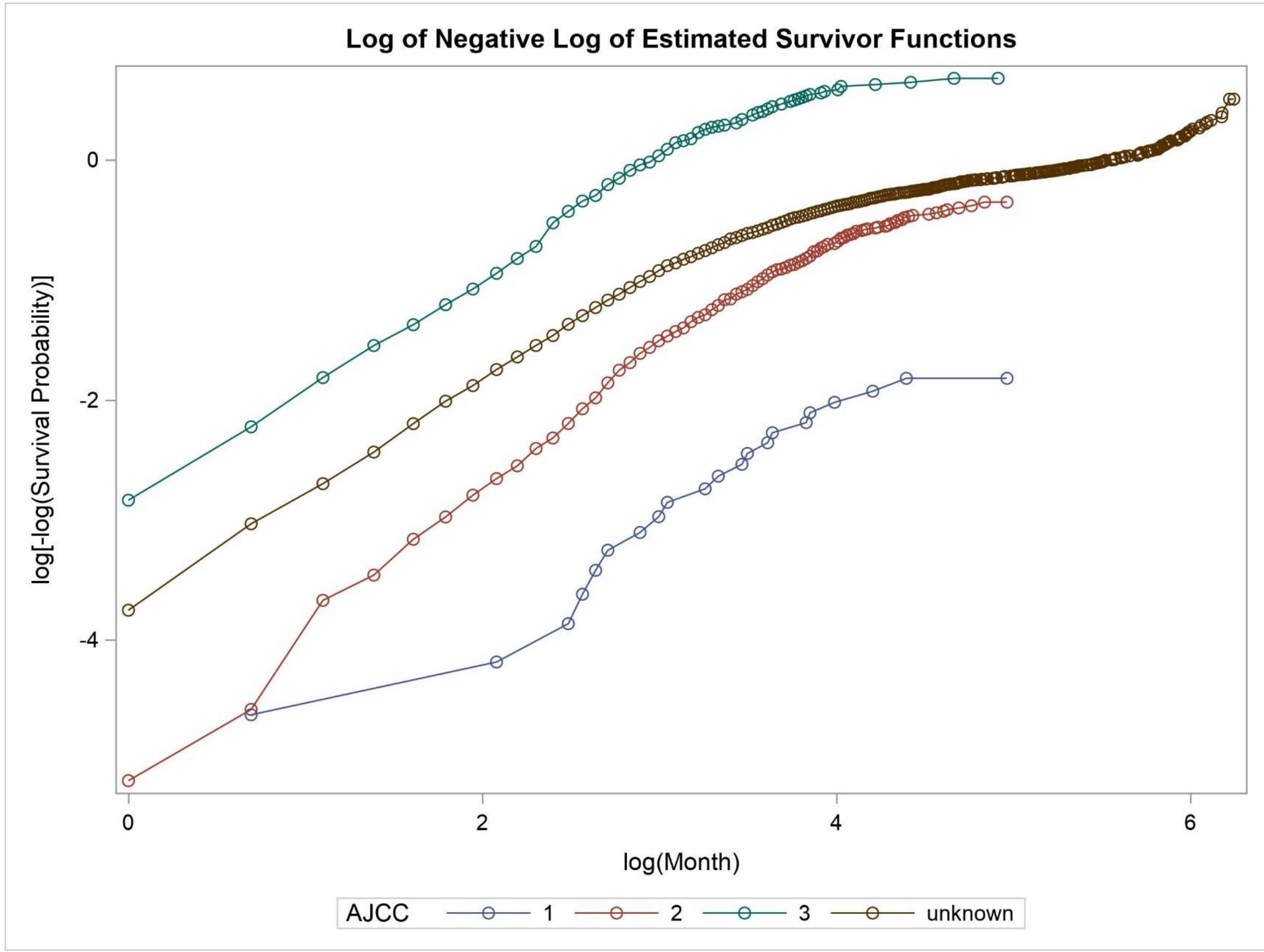

Log of Negative Log of Estimated Survivor Functions
log[-log(Survival Probability)]
0
-2
-4
0
2
4
6
log(Month)
AJCC 1 2 3 unknown

Surgery

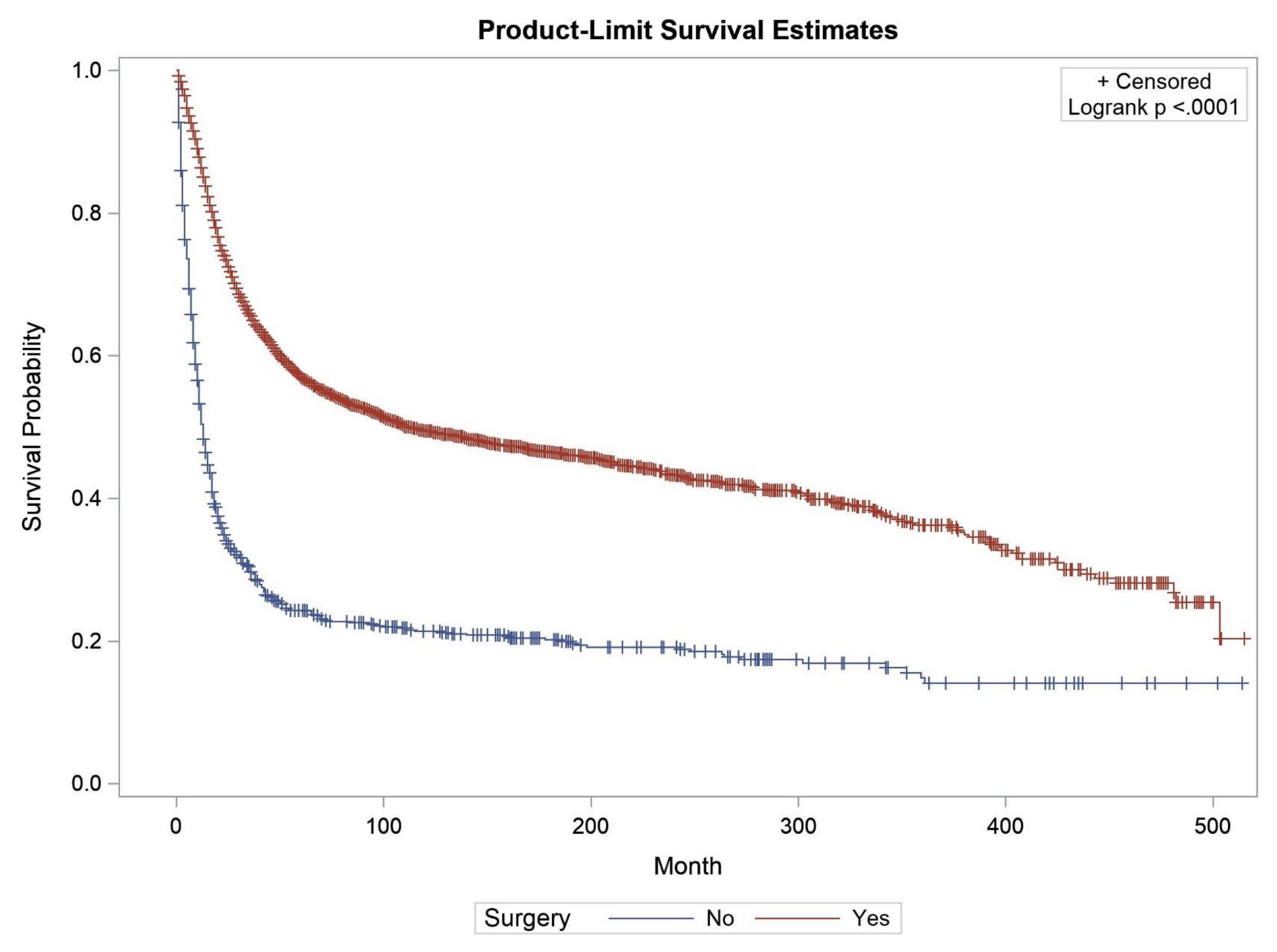

Product-Limit Survival Estimates
+ Censored
Logrank p <.0001
Survival Probability
1.0
0.8
0.6
0.4
0.2
0.0
0
100
200
300
400
500
Month
Surgery
No
Yes

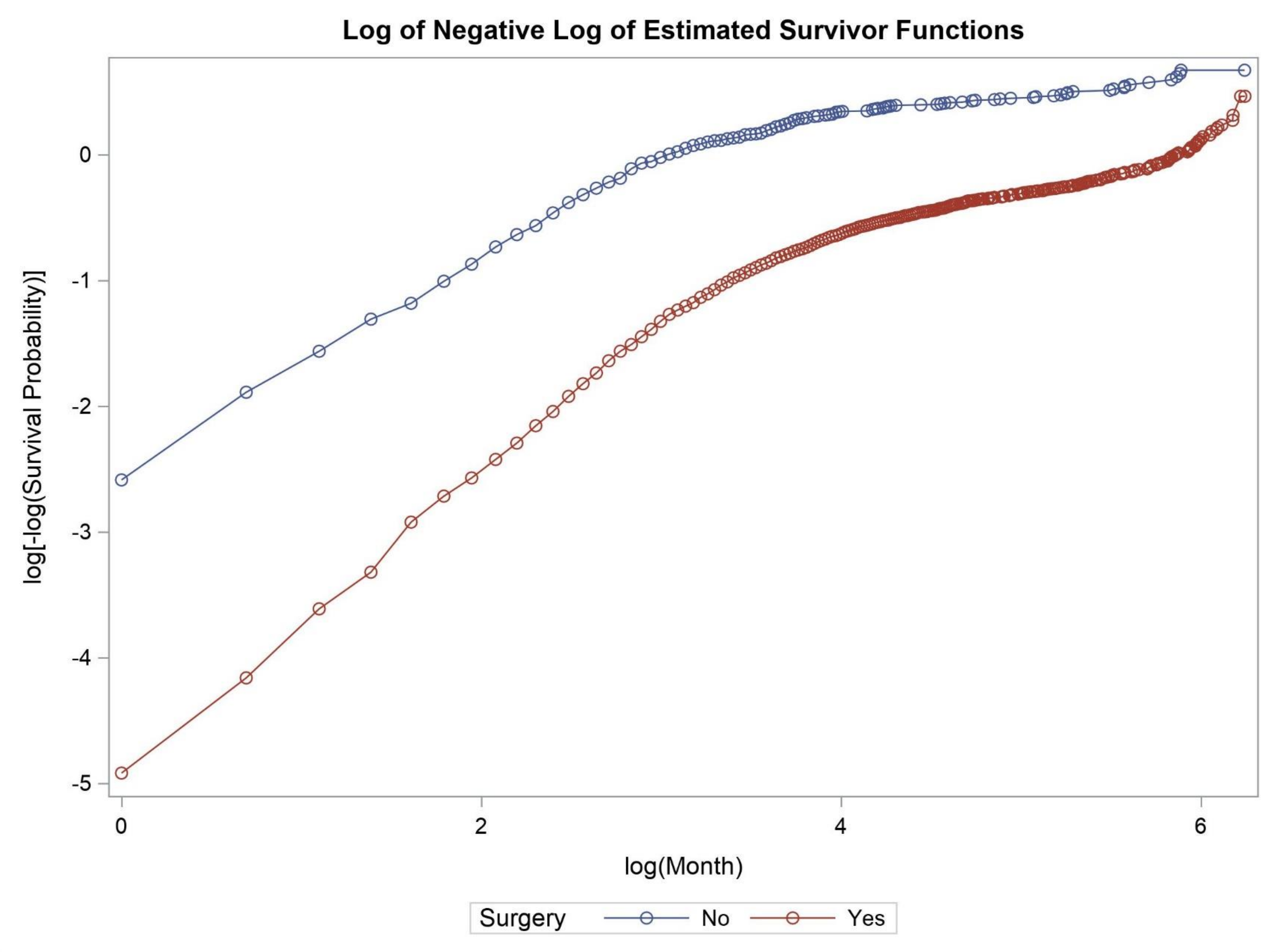

Log of Negative Log of Estimated Survivor Functions
log[-log(Survival Probability)]
0
-1
-2
-3
-4
-5
0
2
4
6
log(Month)
Surgery
No
Yes

Radiation

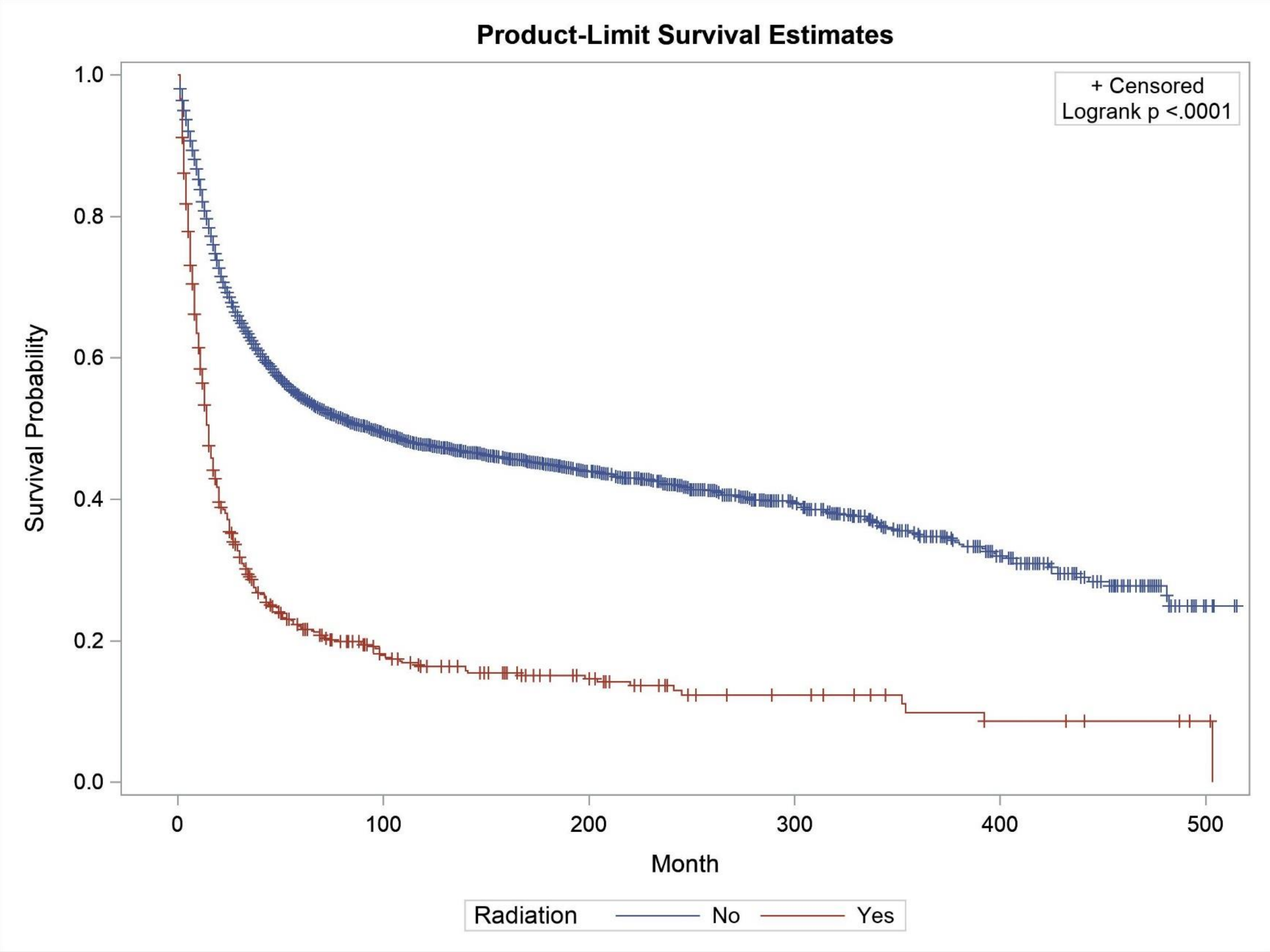
Product-Limit Survival Estimates
+ Censored
Logrank p <.0001
Survival Probability
1.0
0.8
0.6
0.4
0.2
0.0
0
100
200
300
400
500
Month
Radiation
No
Yes

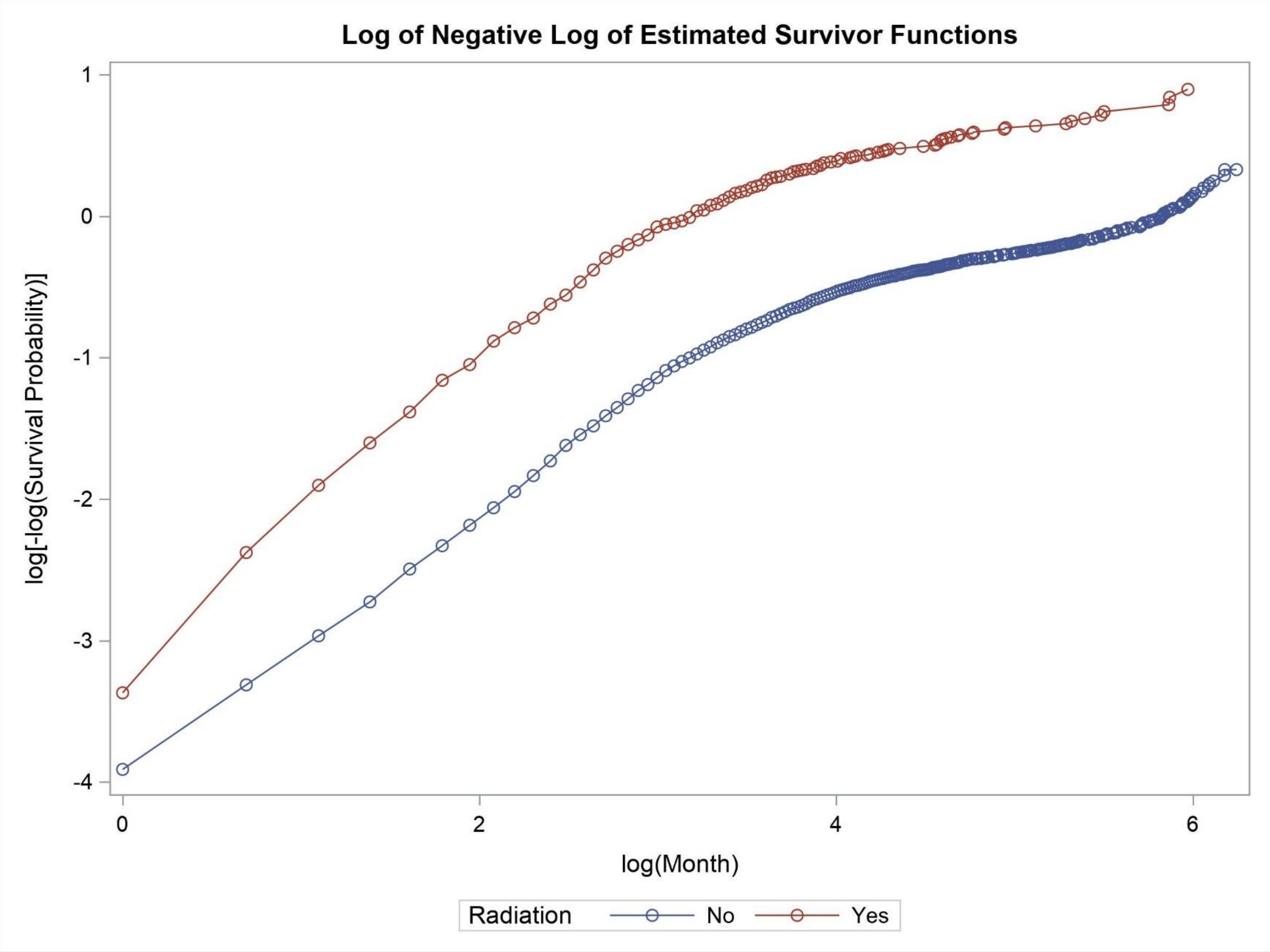
Log of Negative Log of Estimated Survivor Functions
log[-log(Survival Probability)]
1
0
-1
-2
-3
-4
0
2
4
6
log(Month)
Radiation
No
Yes

Chemotherapy

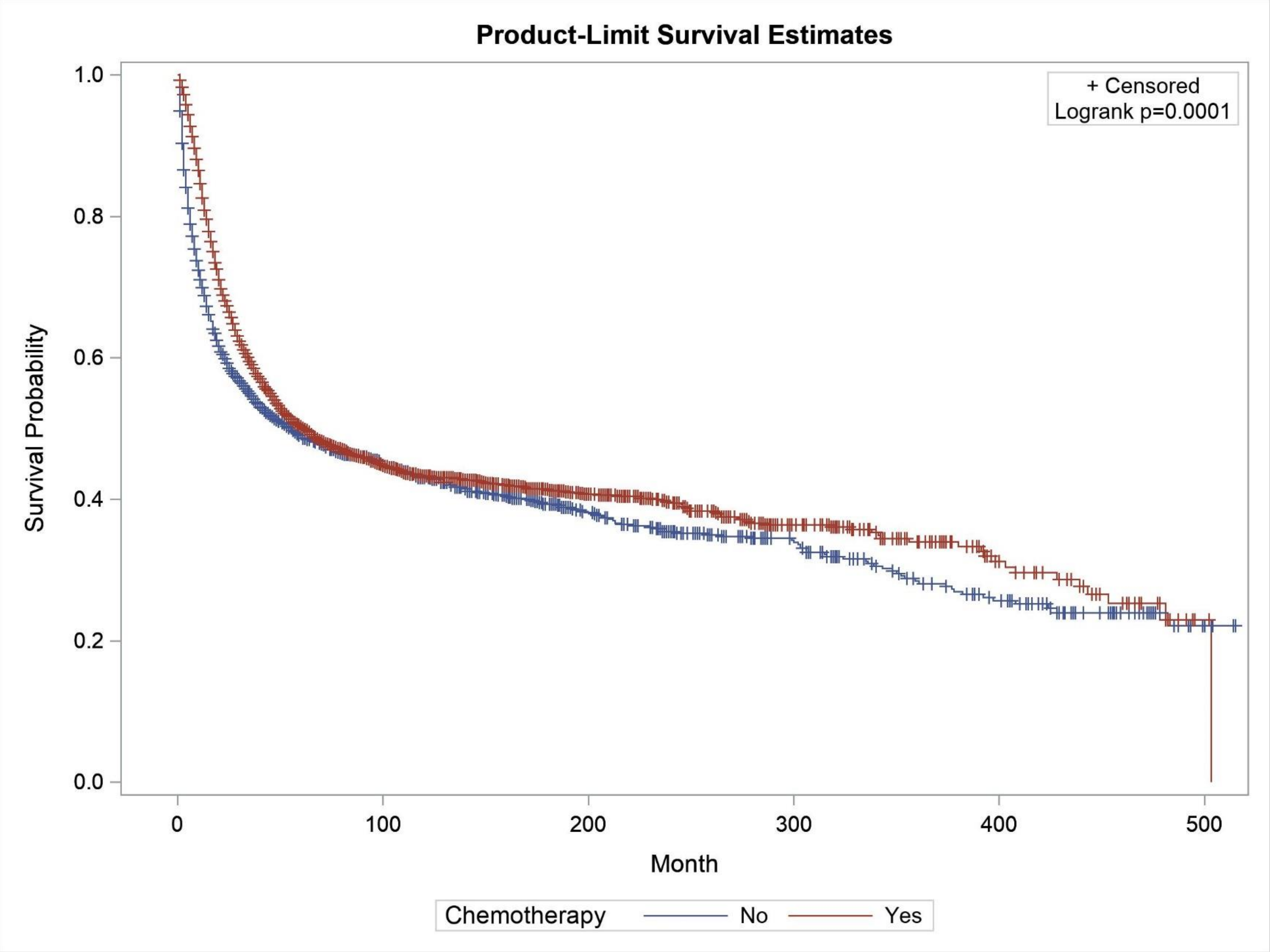

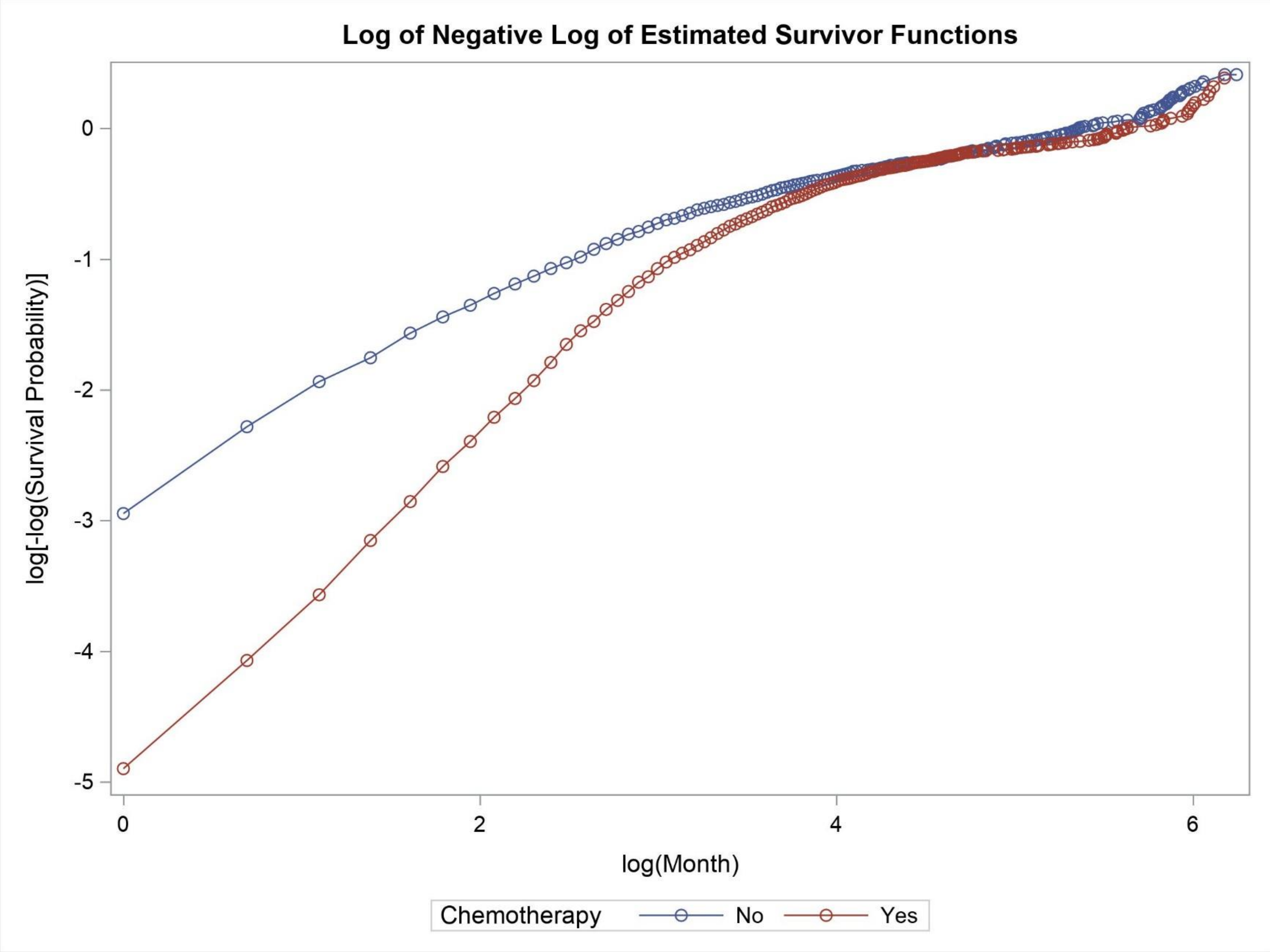

Supplement: Supplementary Materials — Graphic assessment of proportional hazard assumption: graphic assessments (Kaplan-Meier curves and ln (-ln(S(t))) vs. ln(t) Curves) of proportional hazard assumption for included covariates. [file 7264382.f1.pdf]
